# Central metabolism is a key player in *E*. *coli* biofilm stimulation by sub-MIC antibiotics

**DOI:** 10.1371/journal.pgen.1011013

**Published:** 2023-11-02

**Authors:** Luke N. Yaeger, Shawn French, Eric D. Brown, Jean Philippe Côté, Lori L. Burrows

**Affiliations:** 1 Department of Biochemistry and Biomedical Sciences, and the Michael G. DeGroote Institute for Infectious Disease Research, McMaster University, Hamilton, Ontario, Canada; 2 Département de Biologie, Université de Sherbrooke, Sherbrooke, Quebec, Canada; Institut Cochin, FRANCE

## Abstract

Exposure of *Escherichia coli* to sub-inhibitory antibiotics stimulates biofilm formation through poorly characterized mechanisms. Using a high-throughput Congo Red binding assay to report on biofilm matrix production, we screened ~4000 *E*. *coli* K12 deletion mutants for deficiencies in this biofilm stimulation response. We screened using three different antibiotics to identify core components of the biofilm stimulation response. Mutants lacking *acnA*, *nuoE*, or *lpdA* failed to respond to sub-MIC cefixime and novobiocin, implicating central metabolism and aerobic respiration in biofilm stimulation. These genes are members of the ArcA/B regulon–controlled by a respiration-sensitive two-component system. Mutants of *arcA* and *arcB* had a ‘pre-activated’ phenotype, where biofilm formation was already high relative to wild type in vehicle control conditions, and failed to increase further with the addition of sub-MIC cefixime. Using a tetrazolium dye and an *in vivo* NADH sensor, we showed spatial co-localization of increased metabolic activity with sub-lethal concentrations of the bactericidal antibiotics cefixime and novobiocin. Supporting a role for respiratory stress, the biofilm stimulation response to cefixime and novobiocin was inhibited when nitrate was provided as an alternative electron acceptor. Deletion of a gene encoding part of the machinery for respiring nitrate abolished its ameliorating effects, and nitrate respiration increased during growth with sub-MIC cefixime. Finally, in probing the generalizability of biofilm stimulation, we found that the stimulation response to translation inhibitors, unlike other antibiotic classes, was minimally affected by nitrate supplementation, suggesting that targeting the ribosome stimulates biofilm formation in distinct ways. By characterizing the biofilm stimulation response to sub-MIC antibiotics at a systems level, we identified multiple avenues for design of therapeutics that impair bacterial stress management.

## Author summary

Treatment of bacterial pathogens with subinhibitory concentrations of many different types of antibiotics can have the perverse effect of increasing biofilm formation, making infections more difficult to treat and providing opportunities for development of other forms of resistance. The physiological changes that connect subinhibitory antibiotic treatment to increased biofilm are poorly understood. Using *E*. *coli* as a model, we did a genome-wide screen for mutants that failed to make more biofilm when treated with subinhibitory concentrations of three chemically- and mechanistically-distinct antibiotics, looking for commonalities that might highlight key pathways. We identified genes in central metabolism and respiratory pathways, which implicates metabolic changes leading to oxidative stress as important for stimulation, and showed that provision of the alternate electron acceptor nitrate could suppress stimulation for some antibiotics. Bacteriostatic compounds that inhibited translation were uniquely insensitive to nitrate suppression, suggesting that suppression of metabolism can also stimulate biofilm formation. Therefore, divergent perturbations of central metabolism are associated with a similar phenotype of increased biofilm. This work suggests that metabolic changes under antibiotic stress are protective rather than maladaptive as previously thought.

## Introduction

Biofilms are adherent bacterial communities and a common form of bacterial growth [1,2]. They also play a significant role in infection and antibiotic tolerance [3–5]. Biofilms consist of bacterial cells surrounded by a self-produced matrix of exopolysaccharides, extracellular DNA (eDNA), and proteinaceous adhesins that provide protection against physical and chemical threats [6]. The composition of the *Escherichia coli* biofilm matrix varies among strains, but generally includes a combination of curli fimbriae (an amyloid), eDNA, and the exopolysaccharides colanic acid, cellulose, and poly-N-acetyl glucosamine (PNAG) [7–10]. Curli, PNAG, the autotransporter antigen 43, and type 1 fimbriae all contribute to structural integrity of *E*. *coli* biofilms through cell-cell and cell-surface adhesion [7,11–13]. While specific pro-biofilm components drive the emergent properties of biofilms, the production of these components is influenced by an array of central pathways in *E*. *coli* [14]. The *E*. *coli* biofilm life cycle is well-studied, involving reversible and irreversible attachment, maturation, and dispersal [15]. Although biofilms are a common mode of growth, environmental conditions can negatively or positively influence biofilm formation [16].

Treatment with sub-minimal inhibitory concentrations (sub-MIC) of antibiotics stimulates biofilm formation [17]. Biofilms are stimulated by multiple classes of antibiotics, in many different organisms [18], suggesting there are conserved responses to antibiotic stress that converge on increased biofilm formation. The diverse stressors that induce biofilm formation extend beyond antibiotics to type six secretion system effectors, bacteriocins, bacteriophages, and biocides [19–22]. This list of triggers informed the idea of ‘competition sensing’, where cells are proposed to detect damage or kin cell death and respond to protect themselves [23–25]. The induction of biofilms by sub-MIC antibiotics and the ability of biofilms to tolerate multiple stressors suggests that this phenotype falls under the umbrella of competition sensing. Despite the importance of antibiotics, interspecies interactions, and biofilm formation in *E*. *coli* ecology, how antibiotic stress generates a physiological signature that is relayed into increased biofilm formation remains unclear.

We view biofilm stimulation as a defensive response to stressors [17] and consistent with this hypothesis, the major *E*. *coli* stress response pathways Cpx, Rcs, and OmpR influence *E*. *coli* biofilm formation [26–28]. There is also significant input from the stress-responsive sigma factor RpoS and oxidative stress responses in biofilm regulation [14,29,30]. To better understand the mechanism underlying the response to sub-MIC antibiotics, we applied a systems genetics approach to identify genes involved in biofilm stimulation. Here we describe a 1536-colony density, high-throughput screen of the Keio collection–a single gene knockout library of *E*. *coli* K12 [31]–that used uptake of the matrix-binding dye Congo Red as a readout to identify genes important for biofilm stimulation in response to sub-MIC antibiotics of three different classes with three different targets: cefixime (CEF), novobiocin (NOVO), and tetracycline (TET), to identify common pathways. We identified multiple genes in central metabolism and respiration as important for biofilm stimulation in response to each of those drugs. Follow-up experiments also implicated the two-component system ArcA/B in biofilm stimulation, suggesting that oxidative stress from electron transport chain perturbations was a key trigger. We showed that increased respiratory activity spatially colocalizes with and follows treatment by sub-inhibitory antibiotic concentrations. Provision of nitrate in aerobic growth conditions inhibited biofilm stimulation by certain sub-MIC antibiotics, suggesting that an alternative electron acceptor could relieve the oxidative stress that drives stimulation by those compounds. Finally, we linked this observation to nitrate’s role as an electron acceptor using nitrate respiration mutants and a nitrate reduction assay. These results suggest that perturbations of metabolism and respiratory chain activity resulting from exposure to sub-MIC antibiotics drives biofilm stimulation.

## Results

### Development of a high-throughput 1536-colony density biofilm stimulation screen

**Fig 1:**
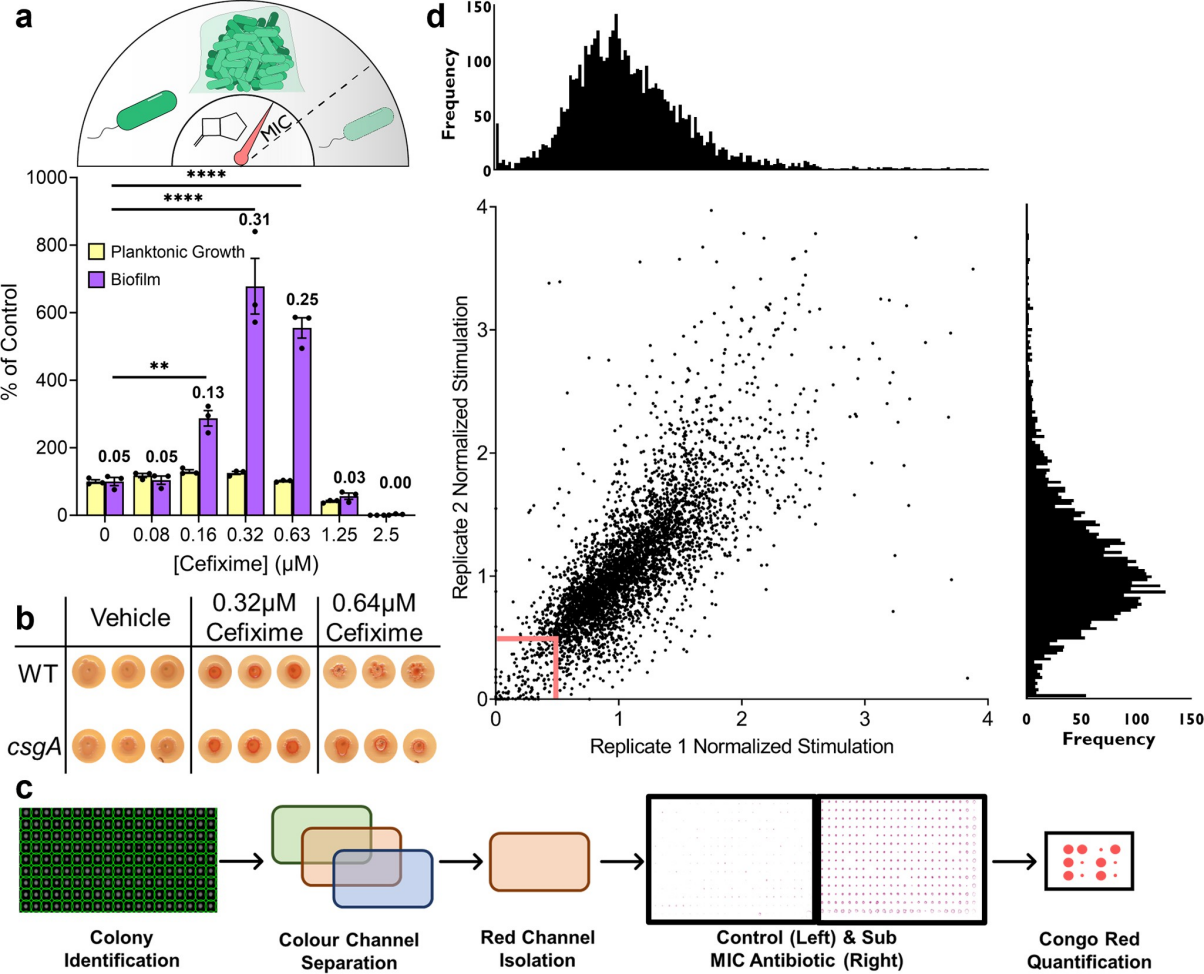
A high throughput screen for genes involved in biofilm stimulation. **a)** (top) An illustration of the biofilm stimulation response, where tuning the levels of antibiotics to just below the MIC stimulates biofilm formation. (bottom) A 96-well peg lid assay to quantify biofilm stimulation in a dose response for *E*. *coli* K12 BW25113. Values are shown as a percent of the vehicle control and each point of a technical triplicate are shown with the bars showing the mean and standard error of the mean shown with lines. Percent of control indicates the Growth (OD_600_) or Biofilm (Abs_600_) values for treatment by a given condition divided by the Growth or Biofilm of the matched vehicle control multiplied by 100. Yellow bars show planktonic growth and purple bars show biofilm. The data are representative of at least 3 biological replicates. The mean Abs_600_ raw values are shown above their respective biofilm bar. A one-way ANOVA followed by Dunnett’s multiple comparisons test was used to compare biofilm formation between the untreated control and CEF treated wells; ** = p value<0.01, **** = p value<0.0001. **b)** Measuring biofilm formation using Congo Red on solid 50:50 agar plates with and without sub-MIC CEF. Colonies are shown for the WT K12 and the *csgA* mutant from the Keio collection **c)** Screening workflow for identifying mutants deficient in biofilm stimulation. **d)** Screening results for CEF shown in a replica plot with extremely high stimulation data points (above 4) removed to help visualize the low stimulation hits. The red box indicates the cut-off for hits, where points within the box indicate hits. Histograms show the distribution of values across each replicate shown on their respective axes.

To identify genes and pathways involved in the *E*. *coli* biofilm stimulation response, we took a forward genetic approach. First, we used a 96-well peg-lid assay [32] to identify a set of antibiotics that elicited a robust biofilm stimulation response at subinhibitory concentrations. The polystyrene peg lid provides a substrate for cell attachment and biofilm formation. Removing the peg lid after incubation allows for separation of adherent from planktonic cells and biofilm quantification. Staining of the peg lids with crystal violet allows for quantification of adhered biomass as a measurement of total biofilm. As expected, biofilm formation peaked at concentrations below those that decreased planktonic growth (**Fig 1A**). From the panel of antibiotics tested (S1 Fig), we selected CEF (cefixime), NOVO (novobiocin), and TET (tetracycline) as model antibiotics with distinct mechanisms of action (targeting peptidoglycan synthesis, DNA replication, and protein synthesis, respectively) that stimulated robust biofilm formation. Although the peg-lid assay is a reproducible method for quantifying biofilms, its relatively low throughput is less amenable to large-scale genetic screens.

To create a colony-based biofilm assay for high-throughput screening, we leveraged the ability of the dye Congo Red (CR) to bind the *E*. *coli* extracellular matrix components curli, cellulose, and PNAG [33,34]. In this assay, the abundance of matrix components serves as a proxy for biofilm formation. We spotted the parent strain of the Keio collection, *E*. *coli* K12 BW25113, on 50:50 LB:PBS agar + CR supplemented with sub-MIC (1/4 to 1/2 MIC) CEF, NOVO, or TET, and grew the plates for 24 h at 37°C–generally considered a suppressive media and temperature for curli expression and CR binding [35,36]. While the resulting colonies on antibiotic-free control plates showed no colour change, those on sub-MIC antibiotic-supplemented plates bound CR and turned red (**Fig 1B**). This result indicates that treatment with sub-MIC antibiotics allows *E*. *coli* to overcome the suppressive effects of the growth conditions on CR binding. Interestingly, curli are likely not solely responsible for antibiotic-induced CR binding, as a *csgA* mutant still showed increased staining upon exposure to sub-MIC antibiotics (**Fig 1B**). Other CR-binding *E*. *coli* biofilm components include cellulose and PNAG; however, K12 strains are unable to produce cellulose [33]. Therefore, the only known CR binding polymer remaining in the *csgA* mutant is PNAG, an exopolysaccharide reported previously to be important for translation inhibitor-induced biofilm formation [37].

We then endeavoured to identify genes important for the physiological changes that precede production of biofilm components. As outlined in **Fig 1C**, the Keio collection was arrayed in 1536-density on CR-supplemented agar plates, first without and with sub-MIC NOVO to optimize the assay conditions, then in a second round of screening, with NOVO, CEF, or TET (below). The plates were grown for 24 h at 37°C, then imaged and analyzed with Fiji [38]. Growth was quantified by measuring colony density; then, the colour channels for each image were separated to isolate the red channel, and the red pixel intensity measured to quantify CR binding. To correct for plate positioning effects, CR binding and growth were normalized by the interquartile mean for each colony’s respective column and row [39]. We generated a CR enrichment value by dividing the normalized CR binding by the normalized growth, and plotted the enrichment values (i.e. normalized stimulation) for both replicates (**Fig 1D**). Mutants with enrichment values at least two standard deviations below the mean in both replicates were classified as hits in our initial NOVO screen. We also identified mutants with enrichment values well above average. These high values could indicate hyper-biofilm stimulation mutants or result from mutations causing small colony sizes, high basal CR binding, or slight changes in antibiotic sensitivity that decreased colony size or impacted the relative MIC to result in more biofilm without affecting a central stress response pathway. For these reasons, we did not further investigate the high enrichment-value mutants as they were more liable to be false positives.

### Central metabolism and respiration genes as major components of biofilm stimulation

**Fig 2:**
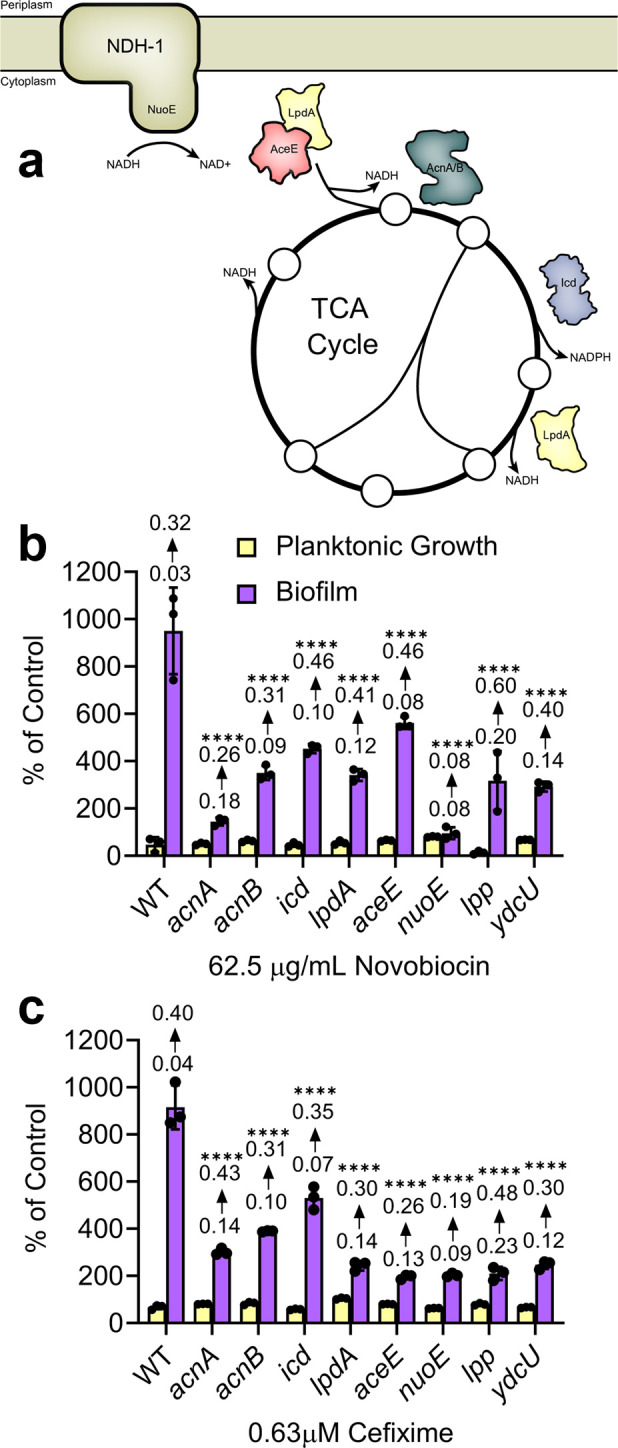
Verification of novobiocin hits using an alternate biofilm assay. **a)** Hits lacking genes involved in central metabolism are illustrated to show their location in the TCA cycle and respiration. Circles indicate metabolites of the TCA cycle. Growth and biofilm formation were measured for cells grown in 50:50 LB with serial dilutions of **b)** NOVO or **c)** CEF using peg-lid assays. Circles on the graphs show data points of technical triplicates that are representative of two biological replicates. Data are shown as a percent of control relative to each mutant’s respective untreated control. Percent of control indicates the Growth (OD_600_) or Biofilm (Abs_600_) values for treatment by a given condition divided by the Growth or Biofilm of the matched vehicle control multiplied by 100. Bars indicate the technical triplicate mean and the error bars show the standard error of the mean. The mean Abs_600_ raw values are shown above their respective biofilm bar, where the bottom value indicates the raw values from the untreated control and the top value indicates the raw values from the antibiotic treated condition. A one-way ANOVA followed by Dunnett’s multiple comparisons test was used to compare biofilm formation between the untreated control and treated wells; **** = p value<0.0001.

We verified loss of biofilm stimulation in response to sub-MIC antibiotics for a subset of the top hits from the screen using a dose-response peg-lid assay. The maximal level of biofilm stimulation for deletion mutants of *acnA* (encoding aconitase), *lpdA* (encoding lipoamide dehydrogenase), *nuoE* (encoding a subunit of complex I in the electron transport chain), *lpp* (encoding Braun’s lipoprotein), and *ydcU* (encoding a predicted polyamine transporter) was markedly reduced upon treatment with either sub-MIC NOVO or CEF compared to the wild type. There is some evidence in the literature that Lpp can indirectly modulate curli synthesis [40], however, the hits were mostly mutants genes related to central metabolism and respiration (**Fig 2A, 2B and 2C**), which we chose to focus on for follow-up experiments. As well, previous studies suggest that antibiotics alter metabolic state and respiration as a part of their lethality [41–45]. Notably, *acnB* is upregulated in response to many different sub-MIC antibiotics [46], while *nuo* mutants were implicated in *E*. *coli* stress-induced mutagenesis via sensing of the ubiquinone pool by the ArcA/B two-component system [47].

To cast a wider net and identify common genes involved in central pathways for biofilm stimulation, we re-screened the entire library with sub-MIC NOVO, CEF, and TET (CEF replica plot shown in **Fig 1D**, NOVO and TET replica plots shown in **S2 Fig**). We selected hits from those screens with normalized enrichment values below 0.5 in both replicates for all three antibiotics. Those with normalized growth that fell below 0.2 were excluded, due to the potential for small colonies to generate false positives. All hits with their predicted functions are listed in **S2 Table** and **S3 Fig** shows a Venn diagram identifying the distribution of shared or unique hits for all three antibiotics.

**Fig 3:**
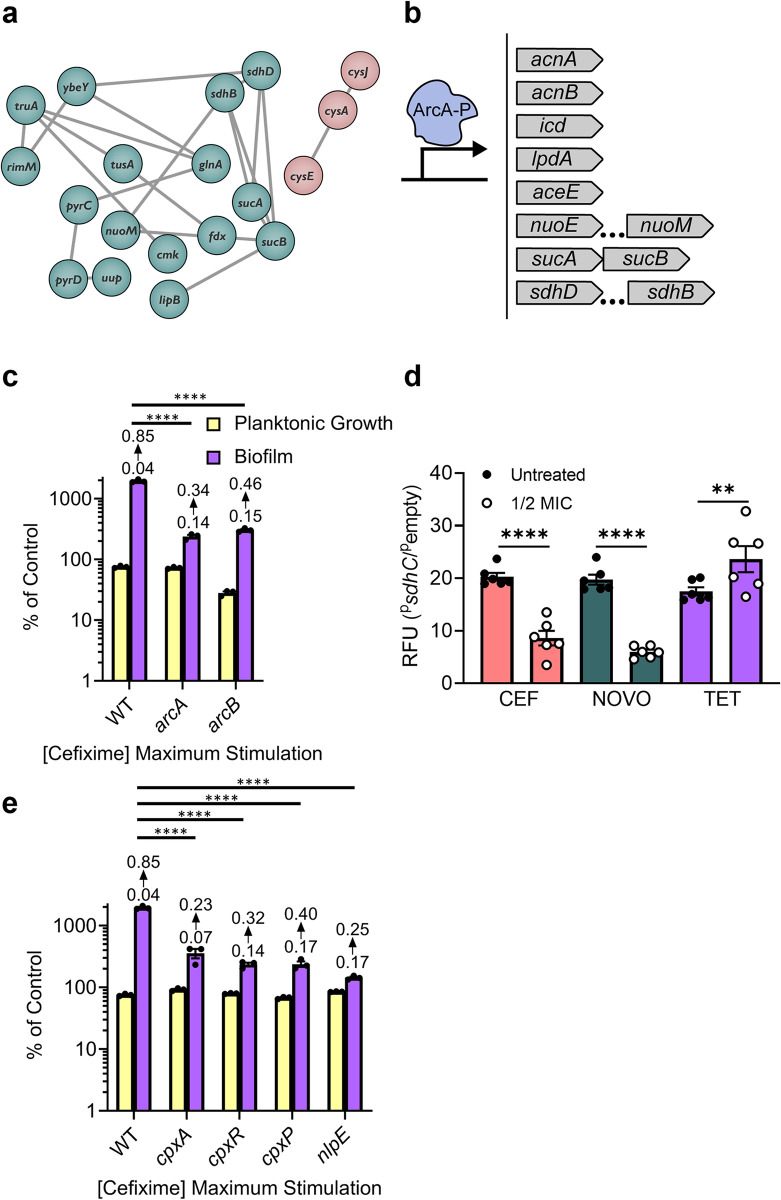
Involvement of metabolic genes and regulatory systems in biofilm stimulation. **a)** A STRING network map of connected hits from the three-drug screen. Connections between each node indicates an experimentally determined interaction, gene fusion, neighbourhood, or co-occurrence, protein homology, co-expression, or text mining. The hits clustered into two groups, indicated by green and pink. The network map was visualized in Cytoscape [114]. **b)** A cartoon depicting the binding of phosphorylated ArcA to a promoter region. The genes from our hit list regulated by ArcA are listed on the right. Ellipses indicate genes in an ArcA-regluated operon that are not directly adjacent. **c)** Biofilm peg-lid assays were done with increasing concentrations of CEF, and only the maximum biofilm stimulation for each strain from these dose-responses is shown here. Planktonic growth and biofilm formation are shown as a percent of the vehicle control. Bars indicate the triplicate mean and the error bars indicate the standard error of the mean. Average background-subtracted crystal violet absorbance values for the untreated controls are shown above each respective biofilm bar. The y-axis is shown as a log scale. A one-way ANOVA followed by Dunnett’s multiple comparisons test was used to calculate statistical significance between the WT and mutant biofilm; **** = p value<0.0001. **d)** Antibiotic-induced changes in activity of the ArcA-P repressed *sdhC* promoter. Changes in relative fluorescence units (RFU) in response to treatment with ½ MIC CEF, NOVO, or TET are shown as bar graphs. All data is relative to the data for the promoter-less plasmid in matched conditions. The bars indicate the mean of six data points from three biological replicates of plates with technical duplicates, the error bars show the standard error of the mean, and the circles indicate each data point value. A one-way ANOVA followed by Dunnett’s multiple comparisons test was used to calculate statistical significance between antibiotic treated and untreated conditions; ** = p value <0.01, **** = p value<0.0001. **e)** Same as for c). For e) and c), percent of control indicates the Growth (OD_600_) or Biofilm (Abs_600_) values for treatment by a given condition divided by the Growth or Biofilm of the matched vehicle control multiplied by 100. As well, the mean Abs_600_ raw values are shown above their respective biofilm bar, where the bottom value indicates the raw values from the untreated control and the top value indicates the raw values from the antibiotic treated condition.

We identified common stimulation-deficient mutants in the lower TCA cycle, (*lipB*, *sucA/B*, *sdhB/D*) that convert 2-oxoglutarate to fumarate while generating NADH for aerobic respiration. A set of genes linked to iron-sulfur cluster biogenesis (*tusA*, *fdx*, *cysJ*, *cysA*) was also identified. Other hits with links to respiration included *nuoM*, *menC*, *pntB*, and *pyrD*. Mutants in ubiquinone biosynthesis were enriched among the hits; however, they had severe growth defects and were filtered from the final list. **Fig 3A** shows a STRING network interaction map for the related hits from the three-drug screen [48]. Together, the data suggest that interrupting flux through the TCA cycle and respiratory chain prevents biofilm stimulation by sub-MIC antibiotic stress. Antibiotic stress was previously shown to perturb central metabolism and aerobic respiration by increasing flux through those pathways [41–45]. In certain conditions, increased flux through the respiratory chain can generate reactive oxygen species (ROS) [49], as electrons stall on solvent-exposed flavins and are transferred to oxygen. Intracellular superoxide and hydrogen peroxide can oxidize iron-sulfur clusters, increasing the free iron available to participate in the Fenton reaction [50,51]. The role of iron in respiration and ROS generation is in line with the data from our screen, connecting the metabolism and iron-sulfur cluster networks shown in **Fig 3A**.

We next considered how *E*. *coli* might sense changes in respiration state [52]. The obvious candidate was ArcA/B, a two-component regulatory system that responds to changes in respiration based on the redox state of the quinone pool, and coordinates expression of metabolism and respiration genes, including many of our hits (**Fig 3B**) [53]. To test the involvement of ArcA/B in the stimulation response, we measured biofilm stimulation for *arcA* and *arcB* mutants (**Fig 3C**). Both had increased baseline biofilm formation (raw crystal violet absorbance values are labelled) and only minimal increases in biofilm levels in response to sub-MIC CEF exposure. These data suggest that inactivation of ArcA/B phenocopies the biofilm stimulation response. It is worth noting that a minimal level of biofilm stimulation was still possible in these mutants, suggesting that additional factors contribute to the stimulation response independent of ArcA/B.

To more directly measure a change in Arc system activity after treatment with sub-MIC antibiotics, we used a promoter reporter for *sdhC*, which is repressed by phosphorylated ArcA [54]. CEF and NOVO increased repression of ^p^*sdhC*, while TET increased promoter activity (**Fig 3D**). This suggested that perhaps CEF and NOVO have opposing effects on Arc activity compared to TET. Notably, TET is a bacteriostatic antibiotic whereas CEF and NOVO are bactericidal.

While the ArcA/B system controls expression of many genes involved in metabolism and respiration, and loss of ArcA reduces competitiveness in a biofilm [55], it does not directly control genes that code for biofilm components. To connect ArcA/B to other systems, we noted previous reports of crosstalk between the Arc and Cpx systems under sub-MIC gentamicin stress [56], so we tested mutants in various steps of the Cpx pathway for biofilm stimulation. The Cpx pathway is responsive to cell envelope stress, affects regulation of respiration complexes, and is implicated in biofilm formation [26,57]. We tested mutants of *cpxA*/*P*/*R* and *nlpE* of the Cpx system. All three *cpx* mutants exhibited reduced biofilm stimulation in response to CEF, while *nlpE* showed no stimulation at all (**Fig 3E**). Further, *cpxR/P* and *nlpE* had elevated baseline biofilm levels. These mutants were not identified in our screen, perhaps due to differences between the CR and peg-lid assays, or changes in antibiotic susceptibility in mutants lacking this system. CpxR/A and NlpE were implicated in *E*. *coli* surface sensing and biofilm formation [26,58,59] and the Raivio group proposed a model in which the Cpx system detects improper assembly of complex I of the electron transport chain (ETC) while also controlling its expression [57,60]. Disrupting the Cpx response could suppress biofilm stimulation by dysregulating maintenance of the ETC.

### Sub-MIC bactericidal antibiotics cause a spike in metabolic activity

**Fig 4:**
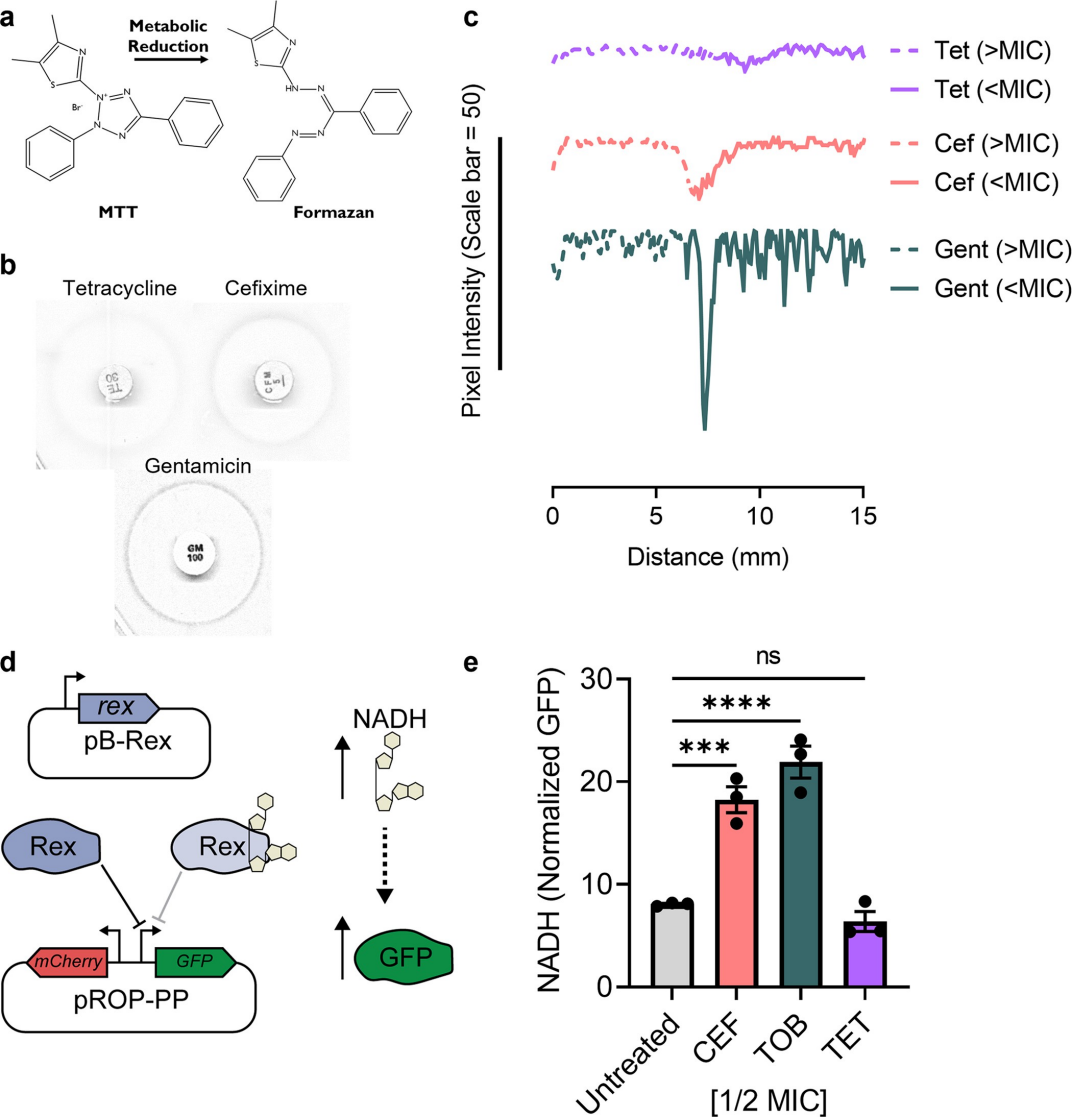
Measuring increased metabolism with sub-MIC antibiotic treatment. **a)** Reduction of the tetrazolium salt MTT into a formazan dye by cellular metabolism. **b)** Images of the zone of inhibition on the MTT agar plates for TET, CEF, and GENT that were used for quantification. **c)** Pixel intensity as a function of distance (in centimeters) from the edge of an antibiotic disk. The zone of inhibition (>MIC) is marked by a dashed lines and extends from the edge of the disk to the point where growth became visible. The sub-MIC zone (<MIC) is marked by solid lines and extends to the edge of the measured region. Data for TET, CEF, and GENT are indicated by purple, red, and green lines, respectively. A scale bar representing 50 units of pixel intensity is shown. The graphs are representative of three biological replicates. **d)** A diagram depicting the synthetic reporter system for *in vivo* NADH detection. Rex is constitutively expressed from pB-Rex and represses expression of GFP from p-ROP-PP-GFP by binding the B-Rex operator site (perfect palindrome). An increase in intracellular NADH leads to more NADH-bound B-Rex and relieves repression of GFP. mCherry is divergently expressed from a constitutive reporter on pROP-PP-GFP to allow for normalization. **e)** Changes in *in vivo* NADH levels as measured by GFP fluorescence are shown as bar graphs for CEF, TOB, or TET treatment. The bars, error bars, and circles represent the technical triplicate mean, standard error of the mean, and individual data points, respectively. A one-way ANOVA followed by Dunnett’s multiple comparisons test was used to compare the untreated and antibiotic treated conditions; ns = not significant, *** = p value <0.001 **** = p value<0.0001.

To visualize the effects of sub-MIC antibiotics on respiration, we used a disc diffusion assay to generate a gradient of antibiotic concentrations. We used a tetrazolium dye, MTT (3-(4,5-dimethylthiazol-2-yl)-2,5-diphenyltetrazolium bromide), that is reduced intracellularly by NADH into a crystalline formazan dye, to monitor changes in cellular respiration (**Fig 4A**) [61]. Formazan dye deposition (indicated by a dark ring and drop in the pixel intensity) clearly localized to the periphery of the zone of inhibition for CEF and GENT, but not TET (**Fig 4B and 4C**). Thus, the bactericidal antibiotics CEF and GENT increase respiration in the sub-MIC zone, whereas the bacteriostatic antibiotic TET does not, consistent with our biofilm data. We also used the two-plasmid *in vivo* NADH reporter system developed by Liu *et al*. to quantify NADH concentration in live cells growing in liquid culture (**Fig 4D**) [62]. As predicted, the bactericidal antibiotics CEF and tobramycin (used in place of GENT as pB-Rex has a GENT resistance cassette) increased NADH levels while TET did not (**Fig 4E**).

### Controlling biofilm stimulation with the terminal electron acceptor nitrate

**Fig 5:**
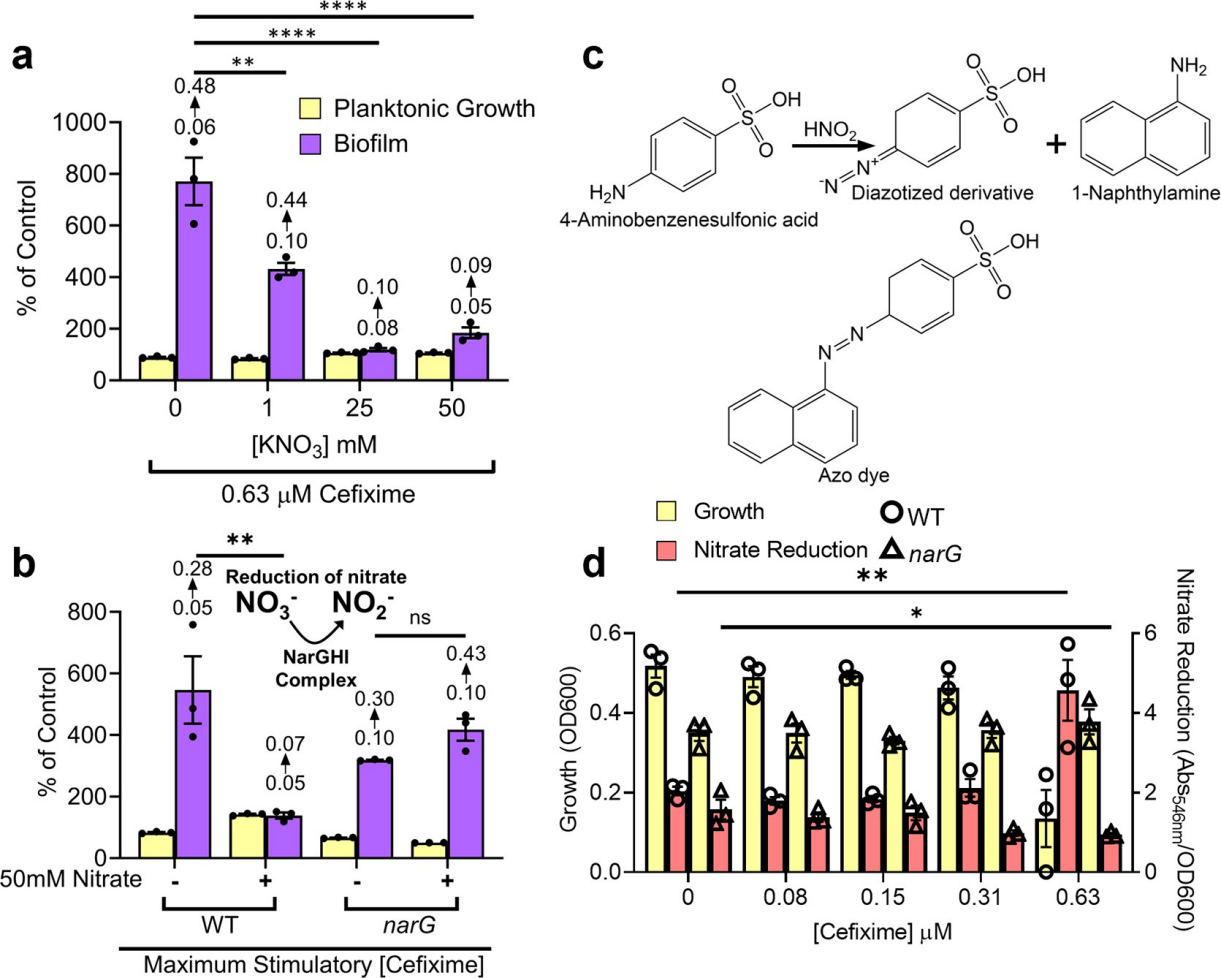
Nitrate respiration suppresses biofilm stimulation. **a)** A dose-response assay showing the effect of nitrate on biofilm stimulation. The data were taken from CEF dose-response peg-lid assays and the maximum biofilm from each nitrate concentration is shown. One representative experiment from three similar biological replicates is shown. A one-way ANOVA followed by a Dunnett’s multiple comparisons test was performed in GraphPad Prism to compare biofilm formation to the no nitrate control (** = p value <0.01, and **** = p value <0.0001) **b)** Effects of nitrate on a nitrate reductase mutant. An illustration of nitrate to nitrite reduction is shown at the top of the panel. Plus and minus signs indicate the presence of 50 mM nitrate in 50:50 broth. The maximum biofilm stimulation from a dose response assay for each strain and condition is shown. Percent of control is relative to the untreated control for each strain and condition. For a) and b), percent of control indicates the Growth (OD_600_) or Biofilm (Abs_600_) values for treatment by a given condition divided by the Growth or Biofilm of the matched vehicle control multiplied by 100. As well, the mean Abs_600_ raw values are shown above their respective biofilm bar, where the bottom value indicates the raw values from the untreated control and the top value indicates the raw values from the antibiotic treated condition. The graph is representative of two biological replicates. A one-way ANOVA followed by Tukey’s multiple comparisons test was performed in GraphPad Prism to compare biofilm formation between the -/+ nitrate wells for the WT and *narG* (** = p value <0.01, and ns = not significant). **c)** The reaction scheme for detecting nitrite from nitrate reduction using a diazotization assay. Nitrite is converted to nitrous acid with acetic acid, which reacts with 4-aminobenzenesulfonic acid to create a diazonium salt that reacts with 1-napthylamine to produce a dye. Chemical structures were created in ChemDraw (PerkinElmer). **d)** Using the diazotization assay from **c)** to measure nitrate reduction after growth of WT (circles) or *narG* (triangles) in increasing sub-MIC CEF concentrations. Planktonic growth is plotted on the left y-axis as the sterility control-subtracted OD_600_ values, and nitrate reduction is shown as the absorbance at 546 nm with sterility control subtracted, divided by the growth of the corresponding well, and plotted on the right y-axis. The graph is representative of two biological replicates. A one-way ANOVA followed by Dunnett’s multiple comparisons test was used to compare nitrate reduction between the treated wells and untreated control for each strain. There was no significant change for any condition except the nitrate reduction for the 0.63μM CEF treated WT and *narG* (* = p value <0.05, ** = p value <0.01). For all graphs, bars indicate the triplicate mean and error bars show the standard error of the mean.

Our data suggest that ETC activity is central to the biofilm stimulation response. Notably, the redox state of ubiquinone/ubiquinol, and the ratio of ubiquinone to menaquinone, which all shuttle electrons through the ETC control ArcA/B activity [53]. We reasoned that if sub-MIC antibiotics were inducing aerobic respiration changes that influenced ArcA/B activity, then supplementing the growth medium with an additional terminal electron acceptor could change the abundance or redox state of quinones to suppress biofilm stimulation. Through these quinones, the ArcA/B system is responsive to the availability of nitrate as an electron acceptor when oxygen availability is restricted [54]. As predicted, potassium nitrate suppressed biofilm stimulation in a dose-dependent manner but without affecting the CEF MIC (**Fig 5A**). To rule out the effect of potassium cations, we tested sodium nitrate, which also inhibited biofilm stimulation (**S4 Fig)**. Notably, nitrate also suppressed biofilm stimulation by NOVO but not TET (**S5 Fig**), which was further explored below. *E*. *coli* dissimilative nitrate respiration follows the ammonification pathway, where nitrate is reduced to nitrite, then ammonia, although the latter step is less frequent due to repression of nitrite reductase by nitrate and efflux of nitrite [63]. Since aerobic conditions such as those in our experiments generally repress nitrate reduction, we controlled for nitrate’s potential ability to inhibit biofilm stimulation through pathways other than respiration. We tested whether a mutant lacking NarG, a subunit of the major nitrate reductase [64], had an altered biofilm stimulation response upon nitrate supplementation. Consistent with our hypothesis that nitrate inhibits biofilm stimulation through its role as an electron acceptor, sub-MIC CEF stimulated biofilm formation by the *narG* mutant, regardless of nitrate addition (**Fig 5B**). We also directly tested if sub-MIC CEF stimulates nitrate reduction using an orthogonal, nitrite-driven diazotization reaction that produces an azo dye (**Fig 5C**) and identified a significant increase in nitrate reduction at ½ MIC (**Fig 5D**) [65,66]. These results are in line with reports of upregulation of anaerobic respiration genes by sub-MIC antibiotics in *Listeria monocytogenes* [67].

### Nitrate insensitivity reveals divergence of biofilm stimulation pathways in response to translation inhibitors

**Fig 6:**
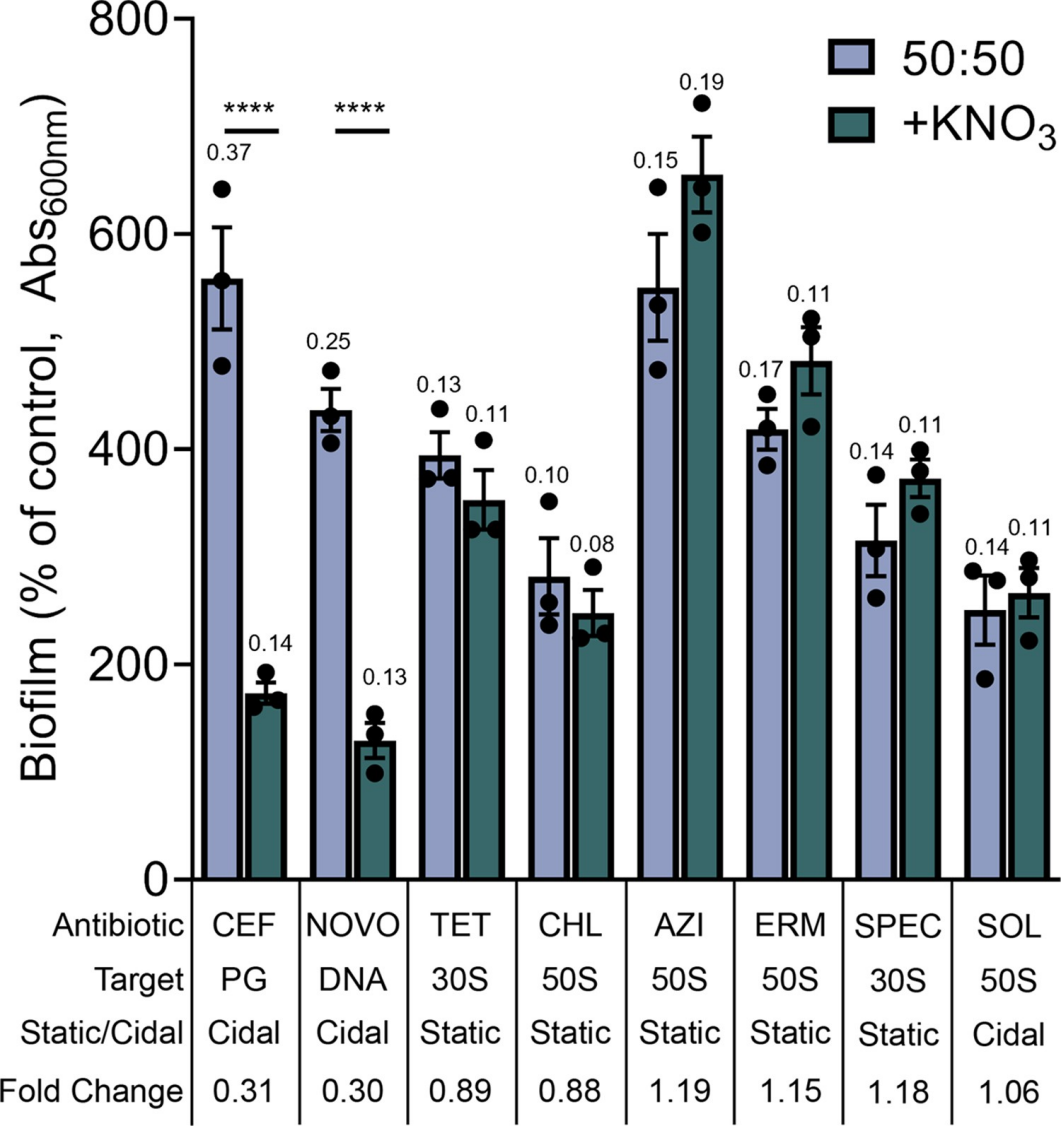
Differing effects of nitrate on biofilm stimulation across a panel of antibiotics. Biofilm assays were performed using peg lids in liquid 50:50 media with or without 50 mM potassium nitrate. Each antibiotic (CEF = cefixime, NOVO = novobiocin, TET = tetracycline, CHL = chloramphenicol, AZI = azithromycin, ERM = erythromycin, SPEC = spectinomycin, SOL = solithromycin) was tested across a range of 2x serial dilutions, and the maximum biofilm stimulation (highest average at one concentration) for each condition was plotted as a percent of the untreated control. Percent of control indicates the Biofilm (Abs_600_) values for treatment by a given condition divided by the Biofilm of the matched vehicle control multiplied by 100. The mean Abs_600_ raw values are shown above their respective biofilm bar. “30S” and “50S” indicate the target subunit for ribosome inhibiting compounds. “Static” and “Cidal” are shorthand for bacteriostatic and bactericidal, respectively. Fold change was calculated by dividing the maximum biofilm stimulation value of the nitrate treated sample by the no nitrate control value. The data are representative of three biological replicates. A one-way ANOVA followed by Šidák’s multiple comparisons test was performed in GraphPad Prism to compare biofilm formation between the -/+ nitrate wells for each antibiotic (**** = p value <0.0001). Unlabelled columns are non-significant.

Nitrate suppressed biofilm stimulation by CEF (**Fig 5**) and NOVO but not TET (**S5 Fig**). To probe the basis of TET insensitivity to nitrate and rule out TET-specific effects, we tested several other translation inhibitors with different features (**Fig 6**). TET acts on the 30S subunit of the ribosome by blocking binding of charged tRNAs to the A site and inhibits translation initiation [68,69]. Chloramphenicol (CHL) targets the 50S subunit at the peptidyl transferase center and inhibits peptide bond formation [69]. Biofilm stimulation in response to CHL was not impacted by nitrate addition, suggesting that lack of nitrate suppression is not specific to 30S targeting antibiotics. CHL activity is context specific, halting translation when an alanine is in the penultimate position on the nascent polypeptide [70,71]. Macrolides azithromycin (AZI) and erythromycin (ERM) target the 50S subunit at the peptide exit tunnel, and also display context-specific inhibition, but for different amino acids than CHL [72]. Further, unlike TET and CHL, AZI and ERM allow for continued translation of some polypeptides [73]. Despite their similar modes of action, AZI and ERM differ in charge, which affects their uptake [74]. The biofilm response to AZI and ERM was unaffected by nitrate addition, indicating that nitrate insensitivity is likely not determined by context-specific inhibition, leaky translation, or charge-dependent uptake. We tested spectinomycin (SPEC), an aminocyclitol antibiotic that targets the 30S subunit and inhibits translocation of tRNAs [69]. Despite a different mechanism of action from the other inhibitors, the biofilm stimulation response to SPEC was also insensitive to nitrate. Finally, we tested solithromycin (SOL), of the ketolide subclass of macrolides that–unlike most in this class–is bactericidal rather than bacteriostatic [75,76]. SOL was a poor biofilm inducer compared to AZI or ERM, so the window for suppression was narrower, but even so, nitrate did not suppress biofilm stimulation. Together, these data suggest that the effect of nitrate does not bisect with the pathway leading to biofilm stimulation upon inhibition of translation.

Given the relatively poor biofilm stimulation by SOL compared to bacteriostatic macrolides, we tested biofilm stimulation by aminoglycosides, bactericidal translation inhibitors that target the 30S subunit. Strikingly, sub-MIC amikacin, streptomycin, and gentamicin (GENT) caused little to no biofilm stimulation (**S6 Fig**). SPEC, which stimulates biofilm well, is structurally similar to aminoglycosides and binds at the same site on the ribosome but is bacteriostatic. This result suggests that cidality and translation inhibition may have opposing impacts on the cellular metabolic cues that lead to biofilm stimulation. Based on these observations, we suggest two possibilities that could explain why bactericidal translation inhibitors are weak biofilm stimulators. First, biofilm stimulation by bactericidal antibiotics requires translation of proteins responsible for increasing biofilm formation; and second, although their cidality increases metabolic demand, translation inhibition stimulates biofilm formation through a pathway involving metabolic dormancy, so the opposing impacts on metabolism effectively cancel one another out.

## Discussion

### Sub-MIC antibiotics drive biofilm stimulation by changing metabolic activity

**Fig 7:**
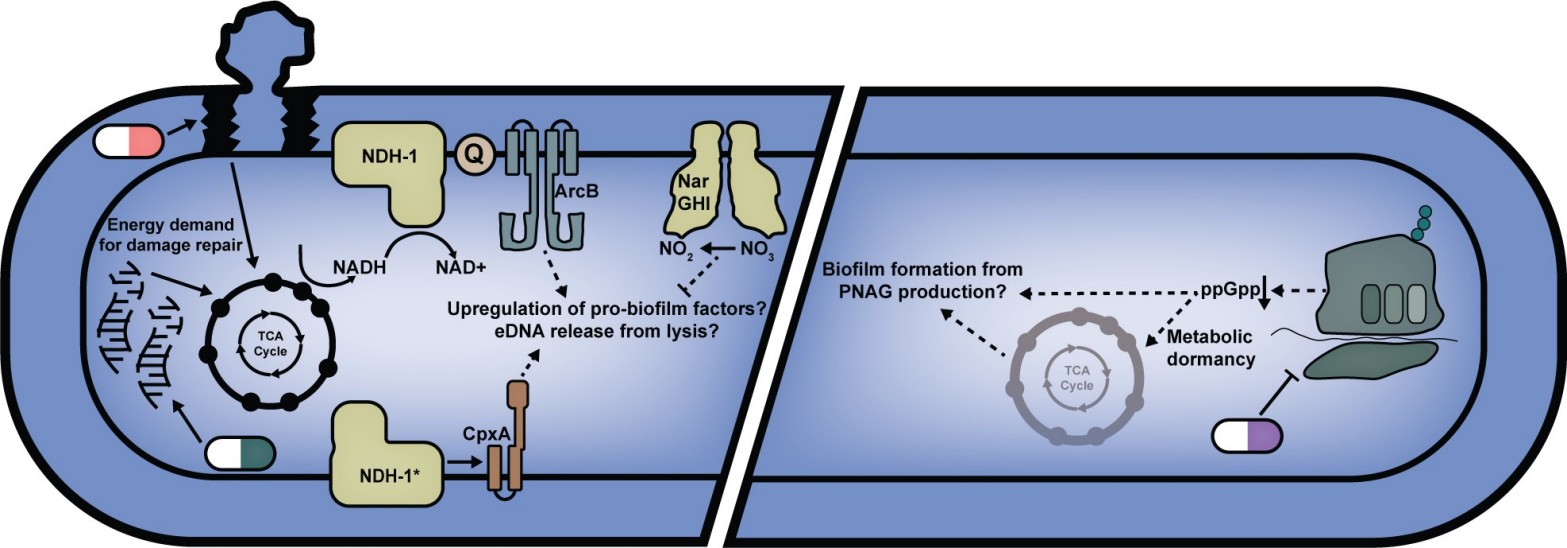
Model of the path from antibiotic stress to biofilm formation. Repairing damage from bactericidal (e.g., cell wall and DNA targeting) antibiotics increases cellular energy demand and consequently flux through the TCA cycle and aerobic respiratory chain. Changes in respiratory chain flux affect the redox status of the quinones (Q) which affects the activity of ArcB [53]. Inner-membrane stress from NDH-1 (complex I) overproduction (indicated by *) activates the Cpx system (based on the Guest et al. 2017 model [57]). Cpx and Arc signalling leads to increased biofilm formation through upregulation of biofilm components and/or increased lysis and eDNA release. Reduction of nitrate by NarGHI suppresses biofilm stimulation. Incorporating our data into the existing model from Boehm et al. (2009) [37], we propose that translation inhibitors induce metabolic dormancy and biofilm formation through reductions in ppGpp.

Using a high-throughput biofilm stimulation screen of the Keio collection, we identified sub-MIC antibiotic-induced metabolism and respiration perturbations as central consequences leading to a subsequent increase in biofilm levels. A connected network of mutants in the lower TCA cycle and respiration were impaired in the biofilm stimulation response to multiple antibiotics. The Arc and Cpx systems that sense respiratory quinone redox state and envelope stress, respectively, were also required for biofilm stimulation (**Fig 7**). Together, the mutants from our screen and follow-up work framed an understanding of the involvement of metabolism and respiration, however future work focusing on individual genes from this set should begin with construction of clean mutants and complementation. For a subset of antibiotics, the stimulation response could be suppressed in a dose- and respiration-dependent manner by the alternate electron acceptor, nitrate. Strikingly, nitrate supplementation had little to no effect on biofilm stimulation by sub-MIC translation inhibitors. Finally, increased metabolic activity colocalized with sub-MIC antibiotic concentrations of bactericidal compounds.

### Biofilm stimulation provides functional context for antibiotic induced metabolic perturbations

We set out to find the common elements in *E*. *coli* physiology that are affected by antibiotics and necessary for biofilm stimulation; re-examining the literature through the lens of our data shows that metabolic and respiratory flux were prime candidates for this role. Metabolic and respiratory changes caused by bactericidal antibiotics are well characterized, particularly by the Collins group [41–46]. Their studies suggest that the lethality of bactericidal antibiotics is driven in part by increased flux through oxidative phosphorylation as a non-optimal response to antibiotic damage. Indeed, peptidoglycan synthesis under beta-lactam stress increases demand for metabolites from glycolysis, causing ROS production [77]. It is curious then, given the lengthy time scale for co-existence of bacteria and antibiotics, that bacteria lack the means to circumvent the harmful increase in flux, although the selective pressure for this may be too low to drive evolution. Based on our data, we propose instead that increased metabolic flux may be advantageous, as increased biofilm formation could protect the population against further antibiotic assault.

Similarly, a network of metabolic genes and the ArcA/B system were implicated in promoting stress-induced mutagenesis [47]. The Arc and the Cpx systems were also implicated in antibiotic cidality [78]. The Collins group found that only bactericidal antibiotics drive increases in metabolic flux, whereas we found that biofilm stimulation can occur in response to both bactericidal and bacteriostatic antibiotics. We showed that the electron acceptor nitrate suppressed biofilm stimulation for the bactericidal cell wall and DNA synthesis-targeting antibiotics CEF and NOVO, but not for the translation inhibitors TET, CHL, AZI, ERM, SPEC, and SOL. This finding suggests that translation inhibitors–unlike other antibiotics–may converge on the biofilm stimulation phenotype through suppressive effects on metabolism (shown on the right of **Fig 7**), supported by our data showing the lack of a metabolic activity spike by cells growing in sub-MIC TET. For example, relative to untreated cells, CHL treatment reduces oxygen consumption rate [41], suggesting that bacteriostatic antibiotics reduce, rather than increase, demand for electron acceptors. The dampening of metabolic demand by bacteriostatic antibiotics is in line with previous work on *E*. *coli* biofilm stimulation by the bacteriostatic translation inhibitors TET and CHL, where biofilm stimulation by these drugs involves de-repression of PNAG biosynthesis through suppression of ppGpp production [37]. Notably, a ppGpp null mutant has decreased expression of many central metabolism genes [79].

From these data, we speculate that bacteriostatic and bactericidal antibiotics may separately converge on biofilm formation through opposing demands on the central metabolism and respiration cues that signal biofilm stimulation. This hypothesis would explain why bactericidal translation inhibitors were poor biofilm stimulators. As well, our screening data showed that NOVO and CEF shared 57 hits that were not hits for TET, while TET shared 26 and six hits with NOVO and CEF, respectively; this result suggests more overlap in the biofilm stimulation response between NOVO and CEF than TET and NOVO or CEF. Of the hits common to NOVO and CEF, there is a notable enrichment of genes involved in energy generation (*menE*, *moaA/C/D/E*, *modA/B/C*, *nuoA/E/I/J/K/N*, *sucD*, and *ubiF*) and related to oxidative stress (*fepB/C/D*, *hscB*, *ybhJ*, and *ygfZ*). The uncoupling of growth inhibition and biofilm stimulation by nitrate could prove to be a useful phenotype for future studies on the direct and downstream effects of bactericidal antibiotics.

### Toward the origin and evolution of biofilm stimulation

A variety of chemically- and mechanistically-distinct antibiotics can induce biofilm formation in a range of bacterial species [17,37,80–86]. Certain antibiotics can inhibit biofilm formation, but these are usually strain or drug-specific effects rather than a generalized response [87–89]. The variety of antibiotics and the number of species demonstrating similar biofilm stimulation phenotypes suggest common, underlying mechanisms of response to antibiotic-induced stresses, rather than reaction to the direct detection of toxic compounds. Boehm *et al*. showed this elegantly with an *E*. *coli* mutant that required the aminoglycoside streptomycin to grow, where biofilm production increased in response to decreasing concentrations of streptomycin [37]. Previous work on biofilm stimulation focused on the roles of specific signalling molecules like cyclic-di-GMP [37,83], ppGpp [37], and quorum sensing molecules [85,86], or eDNA release mechanisms [82,84,86,90,91]. Release of eDNA and bacterial cell death are critical steps in biofilm formation and have many parallels to programmed cell death in eukaryotes [92,93]. Metabolic overflow pathways are important for bacterial programmed cell death and subsequent biofilm development in *S*. *aureus*, in line with our model [94]. It is worth further investigating the parallels between antibiotic-induced biofilm formation and programmed cell death in eukaryotes, as this may shed light on common origins of these pathways [95]. For example, the *P*. *aeruginosa* PQS quorum-sensing derivative HQNO causes cell lysis by disrupting cytochrome *bc1* in the respiratory chain [96], while PQS contributes to biofilm stimulation through an unknown mechanism [86].

### Biofilm stimulation strengthens the connections between respiration status and biofilms

While it is clear that metabolic changes are required for antibiotic-induced biofilm formation, a role for cellular redox state and respiration in normal biofilm formation is well documented [97–99]. Limiting oxidative respiration leads to multicellularity in *Bacillus subtilis* and *S*. *aureus*, which can be rescued by supplying an alternative electron acceptor [100,101]. These responses are mediated by the sensor kinases KinB and SrrB in *B*. *subtilis* and *S*. *aureus*, respectively. In *B*. *subtilis*, KinB increases biofilm formation by phosphorylating the master sporulation regulator Spo0A, while respiration control of biofilm formation in *S*. *aureus* depends on sensing of the menaquinone pool oxidation state by the SrrA/B two-component system and increased transcription of *atlA*, encoding an autolysin that releases eDNA. A *S*. *aureus atlA* mutant is notably unable to respond with increased biofilm formation when exposed to methicillin [84]. In *P*. *aeruginosa*, colony morphogenesis and biofilm formation are influenced by redox state and extracellular electron exchange from phenazines and a cbb3-type oxidase [102,103]. Our data suggest that ArcA/B might function similarly to SrrA/B in sensing changes in respiration status and coordinating biofilm formation in response.

Alternatively, ArcA in *Vibrio cholerae* can be activated by ROS in oxygen-rich conditions that normally inactivate it [104]. We have yet to identify the pro-biofilm effector(s) controlled by ArcA/B, but one possibility for signalling downstream is the Cpx system. Arc and Cpx undergo crosstalk (e.g. phosphorylate the non-cognate regulator) under gentamicin stress [56]. However, if crosstalk was involved in biofilm stimulation, then the presence of ArcB or ArcA alone should be sufficient (either as a phosphodonor or receiver with Cpx), but we did not find this to be the case. The Cpx system is involved in downregulation of cellulose production through OmpA, which causes increased or decreased biofilm formation on hydrophobic or hydrophilic surfaces, respectively [59]. The pathway leading from the Arc/Cpx systems to antibiotic-induced biofilm formation is worth investigating in future work.

Aspects of the biofilm stimulation response have been characterized in select organisms with select antibiotics; however, to our knowledge this is the first systems-level approach focusing on shared components of the response to multiple antibiotics. We found that global changes in central metabolism and respiration connect treatment with sub-MIC antibiotics to increased biofilm formation. This work also provides context for recent studies on antibiotic-induced metabolic changes, as they may represent a general strategy for inducing biofilm formation at sub-MIC levels, to provide subsequent population-level protection against higher concentrations. If increased metabolism/respiration causes a biofilm-promoting protective response, this work would caution against inducing futile cycles as a potential therapeutic strategy [105]. The biofilm stimulation response has also proved useful in our lab as a hypersensitive screen for otherwise-overlooked subinhibitory antibiotic activity [32,106,107].

The work detailed here suggests new possibilities for adjuvant development, detailing targets that, when inhibited, should suppress biofilm stimulation. Mutations in some of the core metabolic genes identified from our screens (*icd*, *sucA*, *nuoM*) that confer antibiotic resistance have been identified in clinical isolates [108], so inhibiting these targets might sensitize cells in addition to suppressing biofilm stimulation. Compounds targeting these metabolic enzymes must avoid inhibition of their eukaryotic homologues, but careful design of narrow spectrum inhibitors of bacterial central metabolism is possible [109,110]. We also showed that nitrate suppresses biofilm stimulation by bactericidal antibiotics. *E*. *coli* is a facultative anaerobe, and regularly encounters oxygen-limited environments when establishing an infection. Although our experiments were performed with a laboratory strain, if the nitrate suppression phenotype is consistent for pathogenic strains, then antibiotic treatment could be coupled with nitrates to prevent the stimulation of biofilm formation. In a similar sense, changes in pH affect the sensitivity of *E*. *coli* to certain beta-lactams [111], and bicarbonate re-sensitizes pathogens to AZI [74], so manipulating host environmental conditions could be a simple and effective strategy to improve antibiotic efficacy. By characterizing the system-level effects of sub-MIC antibiotics on biofilm formation, we have expanded the repertoire of environmental factors and small molecule targets that modulate the response of *E*. *coli* to antibiotic assault.

## Materials and methods

### Bacterial strains, plasmids, and culture conditions

*E*. *coli* studies used the parent strain of the Keio Collection, *E*. *coli* K-12 BW25113 [F−*Δ(araD-araB)567 lacZ4787Δ*::*rrnB-3 LAM− rph-1 Δ(rhaD-rhaB)568 hsdR514*] (denoted herein as *E*. *coli*) [31]. Key mutants selected from the Keio collection were verified with PCR. pB-Rex and pROP-PP-GFP were gifts from Srivatsan Raman (Addgene plasmid #122134; http://n2t.net/addgene:122134; RRID:Addgene_122134, and Addgene plasmid # 122135; http://n2t.net/addgene:122135; RRID:Addgene_122135). For overnight cultures, -80°C stocks were used to inoculate 3 mL of lysogeny broth (LB; Lennox) and incubated at 37°C for 16 h while shaking at 200 rpm. Subcultures were made in 50:50 (50% LB and 50% phosphate buffered saline) and nitrate (KNO_3_ or NaNO_3_) was supplemented where indicated. Nitrate broth (5g/L peptone, 3g/L meat extract, and 50 mM potassium nitrate) was used for the nitrate reduction assay. Plates for solid media assays were made using 1.5% agar and 50:50 media.

### Peg lid biofilm assay

The peg lid biofilm assays were performed as described previously by Ceri et al., with minor modifications [32,112]. Briefly, overnight cultures were diluted 1:100 into a 3 ml subculture of 50:50, grown for ~2 hours and normalized to an OD600 of 0.1 (DeNovix DS-11+ Spectrophotometer), then diluted 1:500 in 50:50. Each well of a flat bottom 96-well plate (Nunc) contained 148 μl of inoculated media and 2 μl of antibiotic/vehicle control. As a sterility control, 148 μl of sterile media and 2 μl of vehicle was pipetted into each well of row H. A peg lid (Nunc) was then set in the plate which was sealed with parafilm. Plates were incubated in a humidity-controlled chamber at 37°C for 18 h while shaking at 200 rpm. After, the peg lid was removed and planktonic growth was measured (OD600, Multiskan Go–Thermo Fisher Scientific). The lid was then washed in phosphate-buffered saline (PBS: 137 mM NaCl, 2.7 mM KCl, 10 mM Na_2_HPO_4_, 1.8 mM KH_2_PO_4_, pH 7.4) and stained in 0.1% (wt/vol) crystal violet for 15 min. Excess crystal violet was removed by 3 separate washes in Milli-Q water and the lids were left to dry. The crystal violet attached to each peg was solubilized in 200 μl of 33% acetic acid (vol/vol) and absorbance was measured at 600 nm (A600, Multiskan Go–Thermo Fisher Scientific). Raw values were plotted as a percent of the vehicle control and statistical analysis was performed using Prism (GraphPadv9.3.1). Each condition was tested in technical triplicate.

### High throughput Congo Red binding screen

For the Keio Collection screen, we poured 25 mL of media (50:50 broth, 1.5% agar, 40 μg/ml Congo Red) supplemented with one of 125 μg/ml novobiocin, 0.6 μM cefixime, or 1 μg/ml tetracycline into rectangular plates. From a source plate, colonies were pinned on the antibiotic-containing plates and once on a vehicle control plate at 1536 colony density using the ROTOR HDA (Singer Instruments). This experiment was done in duplicate using two different source plates. Plates were incubated for 24 h at 37°C, then scanned (Epson Perfection V750) and analyzed with a custom FIJI (ImageJ) program detailed in Appendix I [113]. Data from colonies was normalized by plate position by taking the interquartile mean of each column or row, then dividing each value in that column or row by the interquartile mean. The normalized Congo Red values were divided by the normalized growth values to generate the enrichment value. Mutants with normalized growth below 0.2 were excluded from the hit list. Hits from the first-pass novobiocin screen were manually curated for follow-up validation, while hits from the three antibiotic screen were grouped by GO terms and manually sorted into the major groups listed in S2 Table.

### Promoter reporter assay

Sub-cultures of K12 MG1655 strains containing promoter-less or ^p^*sdhC* pUA66 were grown to an OD600 of 0.1–0.3 in 50:50 supplemented with 50 μg/ml kanamycin, then normalized to 0.1 OD600 and diluted 1:500 into fresh 50:50 supplemented with 50 μg/ml kanamycin. Serial dilutions of cefixime, novobiocin, and tetracycline were added to 96-well plates in technical duplicates with 2x serial dilutions across the rows. Then, 148 μL of the inoculated media was added to each well, excluding the bottom row where sterile media was added. The plates were wrapped with parafilm and incubated at 37°C for 18 h while shaking. After, the OD600 and GFP fluorescence (Ex. 488 nm, Em. 530 nm) were measured with a Synergy Neo (Biotek) plate reader. The average sterility control well values for OD600 and GFP were subtracted from the growth and fluorescence data, respectively, then the GFP values were divided by the OD600 values for each respective well to calculate the relative fluorescence units (RFU). Finally, the ^p^*sdhC* strain values at ½ MIC for each antibiotic were divided by the promoter-less strain values in the antibiotic and concentration-matched wells to obtain a relative change score. The data for each technical duplicate across three biological replicates were plotted using Prism (GraphPadv9.3.1) which was also used for statistical analysis.

### Measuring nitrate reduction

Nitrate reduction was measured as previously described with some modifications to accommodate a 96-well format [65,66]. Briefly, 96-well plates were set up in the same manner as the peg-lid biofilm assay with serial dilutions of antibiotics across the rows, except peg-lids were not added prior to incubation. Strains were sub-cultured in nitrate broth prior to adding to the plate, then transferred in 148 μL of nitrate broth +50mM KNO_3_ for each well then grown for 18 hours at 37°C while shaking, and OD600 was read with a spectrophotometer. Five μL each of nitrate reagent A and B (Sigma) were added to a new 96-well plate, and 100 μL of the culture was added. Absorbance was read at 546 nm immediately after to minimize dye degradation. Raw values were plotted as a percent of the vehicle control and statistical analysis was performed using Prism (GraphPadv9.3.1). Each condition was tested in technical triplicate.

### Disk diffusion MTT reduction

Plates made of 50:50 LB and 1.5% agar were supplemented with 0.04% MTT and dried in a biosafety cabinet. Next, a 10 mL soft LB agar overlay was poured over the plates with the same concentration of MTT, 0.6% agar, and 100 μL of 0.3 OD_600_ *E*. *coli* culture and dried. Once dry, antibiotic disks (Oxoid) were placed on the soft agar overlay with sterile forceps and the plates were incubated for 18 h at 37°C. The plates were then imaged with a flatbed scanner (Canon). ImageJ was used to analyze the resulting images. Prior to analysis, the MTT plate images were split into their RGB colour channels, the green channel was isolated, and the background was subtracted, followed by running the ‘enhance contrast’ function. The ‘plot profile’ function was used to quantify pixel intensity from the edge of the antibiotic disk to the sub-MIC zone just beyond the boundary of the zone of inhibition. Pixel intensity was plotted as a function of distance from the edge of the disk. The data from both plates were graphed using Prism (GraphPadv9.3.1) to compare the pixel intensity profiles of each antibiotic.

### *In vivo* NADH measurement

Overnight cultures of *E*. *coli* K12 BW25113 cells containing the plasmids pB-Rex and pROP-PP-GFP were grown in LB with 15 μg/mL of gentamicin and 50 μg/mL of spectinomycin, then subcultured into 50:50 with the same antibiotic selection. OD600 normalized culture and serial dilutions of cefixime, tetracycline, and tobramycin were added to 96-well plates as for the peg-lid assay. The plate was incubated at 37°C for 18 hours with shaking, then OD600, mCherry fluorescence (Ex. 580 nm, Em. 625 nm), and GFP fluorescence (Ex. 488 nm, Em. 530 nm) were measured with a Synergy Neo (Biotek) plate reader. GFP fluorescence was divided by mCherry fluorescence and OD600 for each well to normalize NADH levels across differences in fluorophore production and growth, respectively.

## Supporting information

S1 Table Strains, Plasmids, and Primers used in this study. (DOCX)

S2 Table Keio screen hits and predicted function common to three sub-MIC antibiotics. (DOCX)

S1 Fig Multiple antibiotics stimulate *E*. *coli* biofilm formation. The effect of increasing concentrations of **a)** NOVO, **b)** TET, **c)** trimethoprim, **d)** polymyxin B, **e)** carbenicillin, **f)** chloramphenicol, and **g)** ciprofloxacin on *E*. *coli* K12 biofilm stimulation was measured using a peg lid assay. All values are relative to the untreated vehicle control for each respective antibiotic. Percent of control indicates the Growth (OD_600_) or Biofilm (Abs_600_) values for treatment by a given condition divided by the Growth or Biofilm of the matched vehicle control multiplied by 100. The mean Abs_600_ raw values are shown above their respective biofilm bar. Circles show the value of each technical replicate of the triplicate, columns show the mean of each replicate, and the bars show the standard error of the mean. Data points are shown as a percent of control, and the y-axis scale on the left applies to all the graphs in-line. A one-way ANOVA followed by Dunnett’s multiple comparisons test was used to calculate statistical significance in biofilm formation between the untreated control and antibiotic treated wells; * = p value<0.05 ** = p value<0.01, *** = p value <0.001 **** = p value<0.0001.
(DOCX)

S2 Fig Biofilm stimulation response to TET and NOVO for the Keio collection. Screening results for TET and NOVO shown in a replica plot with extremely high stimulation data points (above 4) removed to help visualize low stimulation data. The data points inside the red box indicate the hits (below 0.5 CR binding). Screening was done in technical duplicate and data from each replicate were plotted on the x and y-axis.
(DOCX)

S3 Fig Intersection of hits for NOVO CEF or TET. Hits for each antibiotic condition (below 0.5 CR enrichment and above 0.2 growth) were tabulated and input into a custom Venn diagram generator web-tool (http://bioinformatics.psb.ugent.be/webtools/Venn/) to visualize the data. Numbers enumerate genes that were hits for the indicated antibiotic(s).
(DOCX)

S4 Fig Sodium nitrate supplementation suppresses biofilm stimulation. The effect of adding 50 mM sodium nitrate on biofilm stimulation was determined by measuring biofilms with a peg lid assay across a range of CEF concentrations. Data points show the growth and biofilm of each well relative to the respective untreated vehicle control (as a percent of control). Percent of control indicates the Growth (OD_600_) or Biofilm (Abs_600_) values for treatment by a given condition divided by the Growth or Biofilm of the matched vehicle control multiplied by 100. The mean Abs_600_ raw values are shown above their respective biofilm bar. The columns show the mean of each technical triplicate, and the error bars show the standard error of the mean. The data are representative of 3 biological replicates. A two-way ANOVA followed by Šidák’s multiple comparisons test was performed in GraphPad Prism to compare biofilm formation between the -/+ nitrate wells (* = p value<0.05, **** = p value <0.0001). Unlabelled columns are all not significant.
(DOCX)

S5 Fig Effects of providing nitrate on biofilm stimulation by novobiocin and tetracycline. Peg lid biofilm assays were done with increasing concentrations of **a)** NOVO or **b)** TET and either no nitrate (left) or 50 mM nitrate (right). Growth and biofilm formation are shown as a percent of the vehicle control. Percent of control indicates the Growth (OD_600_) or Biofilm (Abs_600_) values for treatment by a given condition divided by the Growth or Biofilm of the matched vehicle control multiplied by 100. The mean Abs_600_ raw values are shown above their respective biofilm bar. Error bars represent the standard error of the mean. Circles show the value of each technical replicate of the triplicate, columns show the mean of each replicate, and the bars show the standard error of the mean. The graphs are representative of 3 biological replicates. A one-way ANOVA followed by Dunnett’s multiple comparisons test was used to calculate statistical significance in biofilm formation between the untreated control and antibiotic treated wells; *** = p value <0.001 **** = p value<0.0001.
(DOCX)

S6 Fig Aminoglycosides are poor stimulators of biofilm formation. Peg lid biofilm assays were performed with increasing concentrations of amikacin, gentamicin, or streptomycin. Growth and biofilm formation are shown as a percent of the vehicle control. Percent of control indicates the Growth (OD_600_) or Biofilm (Abs_600_) values for treatment by a given condition divided by the Growth or Biofilm of the matched vehicle control multiplied by 100. The mean Abs_600_ raw values are shown above their respective biofilm bar. Circles show the value of each technical replicate of the triplicate, columns show the mean of each replicate, and the bars show the standard error of the mean. The graphs are representative of 3 biological replicates. A one-way ANOVA followed by Dunnett’s multiple comparisons test was used to calculate statistical significance in biofilm formation between the untreated control and antibiotic treated wells; none of the antibiotic treatment conditions showed a significant increase in biofilm formation.
(DOCX)

S1 File Annotated ImageJ macro for automated colony analysis (.ijm file) used to process images for colony selection, color channel separation, and measurement of colony density and color intensity. (TXT)

S1 Dataset Normalized CR screening data for 3 antibiotics. Excel file containing raw and normalized screening data.
(XLSX)

S2 Dataset Raw data for Fig 1A. (XLSX)

S3 Dataset Raw data for Fig 2B. (XLSX)

S4 Dataset Raw data for Fig 2C. (XLSX)

S5 Dataset Raw data for Fig 3C and 3E. (XLSX)

S6 Dataset Raw data for Fig 3D. (XLSX)

S7 Dataset Raw data for Fig 4C. (XLSX)

S8 Dataset Raw data for Fig 4E. (XLSX)

S9 Dataset Raw data for Fig 5A. (XLSX)

S10 Dataset Raw data for Fig 5B. (XLSX)

S11 Dataset Raw data for Fig 5D. (XLSX)

S12 Dataset Raw data for Fig 6. (XLSX)

S13 Dataset Raw data for S1 Fig. (XLSX)

S14 Dataset Raw data for S4 Fig. (XLSX)

S15 Dataset Raw data for S5 Fig. (XLSX)

S16 Dataset Raw data for S6 Fig. (XLSX)
