# Supplementary material for: Central metabolism is a key player in E. coli biofilm stimulation by sub-MIC antibiotics
Source: PLoS Genet. 2023 Nov 2;19(11):e1011013. doi: 10.1371/journal.pgen.1011013 (PMC10645362; doi:10.1371/journal.pgen.1011013)
Supplement: S1 Table — (DOCX) [file pgen.1011013.s001.docx]

| **S1 Table. Strains Plasmids, and Primers used in this study.** | | | |
| --- | --- | --- | --- |
| **Strain** | **Accession** | **Description** | **Reference** |
| *E. coli* K-12 BW25113 | N/A | [F−*Δ(araD-araB)567 lacZ4787Δ::rrnB-3 LAM− rph-1 Δ(rhaD-rhaB)568 hsdR514*] | (1) |
| *acnA* | ECK1271 | Aconitase A mutant Kan^R^ | (1) |
| *acnB* | ECK0117 | Aconitase B mutant Kan^R^ | (1) |
| *aceE* | ECK0113 | Pyruvate dehydrogenase mutant Kan^R^ | (1) |
| *icd* | ECK1122 | Isocitrate dehydrogenase mutant Kan^R^ | (1) |
| *nuoE* | ECK2279 | Complex I subunit E mutant Kan^R^ | (1) |
| *lpp* | ECK1673 | Braun’s lipoprotein mutant Kan^R^ | (1) |
| *lpdA* | ECK0115 | Lipoamide dehydrogenase mutant Kan^R^ | (1) |
| *ydcU* | ECK1436 | Putative inner membrane transporter mutant Kan^R^ | (1) |
| *arcA* | ECK4393 | ArcA transcriptional regulator mutant Kan^R^ | (1) |
| *arcB* | ECK3200 | ArcB sensor kinase mutant Kan^R^ | (1) |
| *cpxA* | ECK3904 | CpxA sensor kinase mutant Kan^R^ | (1) |
| *cpxP* | ECK3906 | CpxP periplasmic protein mutant Kan^R^ | (1) |
| *cpxR* | ECK3905 | CpxR transcriptional regulator mutant Kan^R^ | (1) |
| *nlpE* | ECK0192 | Sensor lipoprotein mutant Kan^R^ | (1) |
| *narG* | ECK1218 | nitrate reductase A α subunit mutant Kan^R^ | (1) |
| BW25113 + pB-Rex + pROP-PP-GFP | N/A | *E. coli* K12 BW25113 containing the pB-Rex and p-ROP-PP-GFP vectors to allow for *in vivo* NADH sensing Gent^R^ Spec^R^ | This work |
| *E. coli* K12 MG1655 | N/A | [F-, lambda-, rph-1] | (2) |
| MG1655 + pUA66 (Empty) | N/A | *E. coli* K12 MG1655 containing the pUA66 empty vector Kan^R^ | (2) |
| MG1655 + pUA66 (^p^*sdhC*) | N/A | *E. coli* K12 MG1655 containing the pUA66 vector with the *sdhC* promoter in front of GFP Kan^R^ | (2) |
| **Plasmid** | **Description** | | **Reference** |
| pUA66 (Empty) | Empty vector control from Alon collection containing promoter-less GFP Kan^R^ | | (2) |
| pUA66 (^p^*sdhC*) | Alon collection plasmid containing the *sdhC* promoter upstream of GFP. | | (2) |
| pB-Rex | Constitutive expression of the *Bacillus subtilis* *rex* gene Gent^R^ | | (3) |
| pROP-PP-GFP | Constitutive expression of mCherry and GFP controlled by the perfect palindrome (PP) operator sequence for Rex Spec^R^ | | (3) |
| **Primer** | **Sequence (5’-3’)** | | |
| *acnA*-Fwd | TCGTCAACCCTACGAGAAGCCA | | |
| *acnA*-Rvs | CTTCAACATATTACGAATGACATAATGCAAAATGCCG | | |
| *acnB*-Fwd | CTAGAAGAATACCGTAAGCACGTAGCTGAG | | |
| *acnB*-Rvs | AACCGCAGTCTGGAAAATCACCC | | |
| *narG*-Fwd | AGTAAATTCCTGGACCGGTTTCGCTAC | | |
| *narG*-Rvs | TTTTACGCTCTCCTGTACCTGGTCATTG | | |
| *lpp*-Fwd | AAAGCTACTAAACTGGTACTGGGCGC | | |
| *lpp*-Rvs | CTTGCGGTATTTAGTAGCCATGTTGTCCA | | |
| *arcA*-Fwd | CAGACCCCGCACATTCTTATCGTTGAA | | |
| *arcA*-Rvs | ATCTTCCAGATCACCGCAGAAGCG | | |
| *arcB*-Fwd | AAGCAAATTCGTCTGCTGGCG | | |
| *arcB*-Rvs | TTTTTTAGTGGCTTTTGCCACCCACG | | |
| FRT-site specific | GAACTGCAGGTCGACGGATCC | | |

**Supplemental References for S1 Table:**

1. Baba T, Ara T, Hasegawa M, Takai Y, Okumura Y, Baba M, Datsenko KA, Tomita M, Wanner BL, Mori H. 2006. Construction of *Escherichia coli* K-12 in-frame, single-gene knockout mutants: The Keio collection. Mol Syst Biol 2:2006.0008.

2. Zaslaver A, Bren A, Ronen M, Itzkovitz S, Kikoin I, Shavit S, Liebermeister W, Surette MG, Alon U. 2006. A comprehensive library of fluorescent transcriptional reporters for *Escherichia coli*. Nat Methods 3:623–628.

3. Liu Y, Landick R, Raman S. 2019. A Regulatory NADH/NAD+ Redox Biosensor for Bacteria. ACS Synth Biol 8:264–273.
