# Supplementary material for: Central metabolism is a key player in E. coli biofilm stimulation by sub-MIC antibiotics
Source: PLoS Genet. 2023 Nov 2;19(11):e1011013. doi: 10.1371/journal.pgen.1011013 (PMC10645362; doi:10.1371/journal.pgen.1011013)
Supplement: S2 Table — (DOCX) [file pgen.1011013.s002.docx]

| **S2 Table. Keio screen hits and predicted function common to three sub-MIC antibiotics.** | | |
| --- | --- | --- |
| **Gene** | **Predicted Function** | **Reference** |
| **Respiration and Central Metabolism** |  |  |
| *sucA* | Succinyl-CoA synthesis | (1) |
| *sucB* | Succinyl-CoA synthesis | (1) |
| *sdhB* | Succinate:quinone oxidoreductase | (2) |
| *sdhD* | Succinate:quinone oxidoreductase | (2) |
| *nuoM* | NADH:quinone oxidoreductase | (3) |
| *lipB* | Lipoyl transferase | (4) |
| *menC* | Menaquinone biosynthesis | (5) |
| *pntB* | NAD phosphorylation | (6) |
| **Sulfur Metabolism** |  |  |
| *cysE* | Serine acetyltransferase/cysteine biosynthesis | (7) |
| *tusA* | Sulfur transfer protein | (8) |
| *fdx* | Fe-S cluster assembly | (9) |
| *cysJ* | Sulfite reductase | (10) |
| *cysA* | Sulfate/thiosulfate ABC transporter | (11) |
| **Nucleotide Metabolism/Transcription and Translation** |  |  |
| *cmk* | Cytidylate kinase | (12) |
| *yegP* | Double stranded break repair | (13) |
| *pyrC* | Dihydroorotase/pyrimidine biosynthesis | (14) |
| *pyrD* | Dihydroorotate dehydrogenase/pyrimidine biosynthesis | (15) |
| *uup* | ATP-binding protein/transposon excision | (16) |
| *truA* | tRNA pseudouridine synthase | (17) |
| *ybeY* | Endoribonuclease/16s rRNA maturation | (18) |
| *rimM* | 30s ribosomal subunit assembly | (19) |
| **Miscellaneous** |  |  |
| *glnA* | Glutamine synthetase | (20) |
| *nhaA* | Na^+^:K^+^ antiporter | (21) |
| *gmhB* | D,D-heptose 1,7-bisphosphate phosphatase | (22) |
| *lon* | Protease | (23) |
| *yggT* | Osmotic regulation | (24) |
| *gntK* | D-gluconate kinase | (25) |
| *yehP* | Swarming motility (VWA domain-containing protein) | (26) |
| *ydgU* | Small, uncharacterized membrane protein | (27) |
| *fes* | Enterobactin hydrolysis | (28) |

**Supplemental references for S2 Table:**

1. Buck D, Spencer ME, Guest JR. 1986. Cloning and expression of the succinyl-CoA synthetase genes of *Escherichia coli* K12. J Gen Microbiol 132:1753–62.

2. Cecchini G, Schröder I, Gunsalus RP, Maklashina E. 2002. Succinate dehydrogenase and fumarate reductase from *Escherichia coli*. Biochim Biophys Acta - Bioenerg https://doi.org/10.1016/S0005-2728(01)00238-9.

3. Erhardt H, Steimle S, Muders V, Pohl T, Walter J, Friedrich T. 2012. Disruption of individual *nuo* genes leads to the formation of partially assembled NADH:ubiquinone oxidoreductase (complex I) in *Escherichia coli*. Biochim Biophys Acta - Bioenerg 1817:863–871.

4. Reed KE, Cronan JE. 1993. Lipoic acid metabolism in *Escherichia coli*: Sequencing and functional characterization of the *lipA* and *lipB* genes. J Bacteriol 175:1325–1336.

5. Sharma V, Meganathan R, Hudspeth MES. 1993. Menaquinone (vitamin K2) biosynthesis: Cloning, nucleotide sequence, and expression of the *menC* gene from *Escherichia coli*. J Bacteriol 175:4917–4921.

6. Ahmad S, Glavas NA, Bragg PD. 1992. Subunit interactions involved in the assembly of pyridine nucleotide transhydrogenase in the membranes of *Escherichia coli*. J Biol Chem 267:7007–12.

7. Denk D, Böck A. 1987. L-cysteine biosynthesis in *Escherichia coli*: nucleotide sequence and expression of the serine acetyltransferase (*cysE*) gene from the wild-type and a cysteine-excreting mutant. J Gen Microbiol 133:515–25.

8. Ikeuchi Y, Shigi N, Kato JI, Nishimura A, Suzuki T. 2006. Mechanistic insights into sulfur relay by multiple sulfur mediators involved in thiouridine biosynthesis at tRNA wobble positions. Mol Cell 21:97–108.

9. Kakuta Y, Horio T, Takahashi Y, Fukuyama K. 2001. Crystal structure of *Escherichia coli* Fdx, an adrenodoxin-type ferredoxin involved in the assembly of iron-sulfur clusters. Biochemistry 40:11007–11012.

10. Eschenbrenner M, Coves J, Fontecave M. 1995. The flavin reductase activity of the flavoprotein component of sulfite reductase from *Escherichia coli*. A new model for the protein structure. J Biol Chem 270:20550–20555.

11. Sirko A, Hryniewicz M, Hulanicka D, Bock A. 1990. Sulfate and thiosulfate transport in *Escherichia coli* K-12: Nucleotide sequence and expression of the *cysTWAM* gene cluster. J Bacteriol 172:3351–3357.

12. Ofiteru A, Bucurenci N, Alexov E, Bertrand T, Briozzo P, Munier-Lehmann H, Gilles AM. 2007. Structural and functional consequences of single amino acid substitutions in the pyrimidine base binding pocket of *Escherichia coli* CMP kinase. FEBS J 274:3363–3373.

13. Kumar A, Beloglazova N, Bundalovic-Torma C, Phanse S, Deineko V, Gagarinova A, Musso G, Vlasblom J, Lemak S, Hooshyar M, Minic Z, Wagih O, Mosca R, Aloy P, Golshani A, Parkinson J, Emili A, Yakunin AF, Babu M. 2016. Conditional Epistatic Interaction Maps Reveal Global Functional Rewiring of Genome Integrity Pathways in *Escherichia coli*. Cell Rep 14:648–661.

14. Porter TN, Li Y, Raushel FM. 2004. Mechanism of the dihydroorotase reaction. Biochemistry 43:16285–16292.

15. Fagan RL, Palfey BA. 2009. Roles in binding and chemistry for conserved active site residues in the class 2 dihydroorotate dehydrogenase from *Escherichia coli*. Biochemistry 48:7169–7178.

16. Zepeda MYB, Alessandri K, Murat D, El Amri C, Dassa E. 2010. C-terminal domain of the Uup ATP-binding cassette ATPase is an essential folding domain that binds to DNA. Biochim Biophys Acta - Proteins Proteomics 1804:755–761.

17. Kammen HO, Marvel CC, Hardy L, Penhoet EE. 1988. Purification, structure, and properties of *Escherichia coli* tRNA pseudouridine synthase I. J Biol Chem 263:2255–63.

18. Jacob AI, Köhrer C, Davies BW, RajBhandary UL, Walker GC. 2013. Conserved Bacterial RNase YbeY Plays Key Roles in 70S Ribosome Quality Control and 16S rRNA Maturation. Mol Cell 49:427–438.

19. Guo Q, Goto S, Chen Y, Feng B, Xu Y, Muto A, Himeno H, Deng H, Lei J, Gao N. 2013. Dissecting the in vivo assembly of the 30S ribosomal subunit reveals the role of RimM and general features of the assembly process. Nucleic Acids Res 41:2609–2620.

20. Colombo G, Villafranca JJ. 1986. Amino acid sequence of *Escherichia coli* glutamine synthetase deduced from the DNA nucleotide sequence. J Biol Chem 261:10587–91.

21. Appel M, Hizlan D, Vinothkumar KR, Ziegler C, Kühlbrandt W. 2009. Conformations of NhaA, the Na+/H+ exchanger from *Escherichia coli*, in the pH-activated and ion-translocating states. J Mol Biol https://doi.org/10.1016/j.jmb.2009.03.010.

22. Kneidinger B, Marolda C, Graninger M, Zamyatina A, McArthur F, Kosma P, Valvano MA, Messner P. 2002. Biosynthesis pathway of ADP-L-glycero-β-D-manno-heptose in *Escherichia coli*. J Bacteriol 184:363–369.

23. Botos I, Melnikov EE, Cherry S, Tropea JE, Khalatova AG, Rasulova F, Dauter Z, Maurizi MR, Rotanova T V, Wlodawer A, Gustchina A. 2004. The Catalytic Domain of *Escherichia coli* Lon Protease Has a Unique Fold and a Ser-Lys Dyad in the Active Site. J Biol Chem 279:8140–8148.

24. Ito T, Uozumi N, Nakamura T, Takayama S, Matsuda N, Aiba H, Hemmi H, Yoshimura T. 2009. The implication of *yggT* of *Escherichia coli* in osmotic regulation. Biosci Biotechnol Biochem 73:2698–2704.

25. Izu H, Adachi O, Yamada M. 1996. Purification and characterization of the *Escherichia coli* thermoresistant gluconokinase encoded by the *gntK* gene. FEBS Lett 394:14–16.

26. Inoue T, Shingaki R, Hirose S, Waki K, Mori H, Fukui K. 2007. Genome-wide screening of genes required for swarming motility in *Escherichia coli* K-12. J Bacteriol 189:950–957.

27. Hemm MR, Paul BJ, Schneider TD, Storz G, Rudd KE. 2008. Small membrane proteins found by comparative genomics and ribosome binding site models. Mol Microbiol 70:1487–1501.

28. Winkelmann G, Cansier A, Beck W, Jung G. 1994. HPLC separation of enterobactin and linear 2,3-dihydroxybenzoylserine derivatives: a study on mutants of *Escherichia coli* defective in regulation (*fur*), esterase (*fes*) and transport (*fepA*). Biometals 7:149–54.
