# Supplementary material for: Central metabolism is a key player in E. coli biofilm stimulation by sub-MIC antibiotics
Source: PLoS Genet. 2023 Nov 2;19(11):e1011013. doi: 10.1371/journal.pgen.1011013 (PMC10645362; doi:10.1371/journal.pgen.1011013)
Supplement: S1 Fig — The effect of increasing concentrations of a) NOVO, b) TET, c) trimethoprim, d) polymyxin B, e) carbenicillin, f) chloramphenicol, and g) ciprofloxacin on E. coli K12 biofilm stimulation was measured using a peg lid assay. All values are relative to the untreated vehicle control for each respective antibiotic. Percent of control indicates the Growth (OD600) or Biofilm (Abs600) values for treatment by a given condition divided by the Growth or Biofilm of the matched vehicle control multiplied by 100. The mean Abs600 raw values are shown above their respective biofilm bar. Circles show the value of each technical replicate of the triplicate, columns show the mean of each replicate, and the bars show the standard error of the mean. Data points are shown as a percent of control, and the y-axis scale on the left applies to all the graphs in-line. A one-way ANOVA followed by Dunnett’s multiple comparisons test was used to calculate statistical significance in biofilm formation between the untreated control and antibiotic treated wells; * = p value<0.05 ** = p value<0.01, *** = p value <0.001 **** = p value<0.0001. (DOCX) [file pgen.1011013.s003.docx]

**
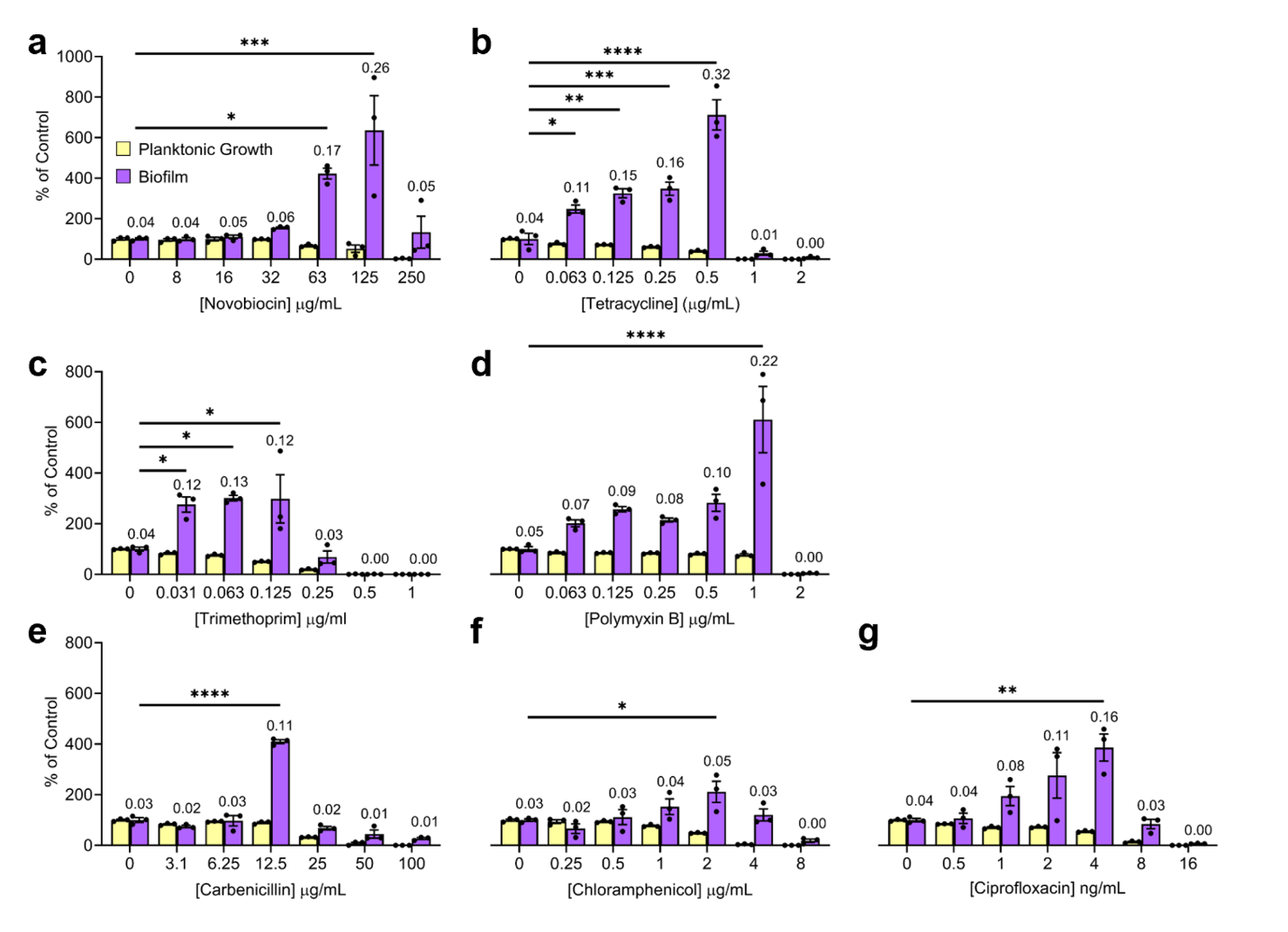
**

**S1 Fig. Multiple antibiotics stimulate *E. coli* biofilm formation.** The effect of increasing concentrations of **a)** NOVO, **b)** TET, **c)** trimethoprim, **d)** polymyxin B, **e)** carbenicillin, **f)** chloramphenicol, and **g)** ciprofloxacin on *E. coli* K12 biofilm stimulation was measured using a peg lid assay. All values are relative to the untreated vehicle control for each respective antibiotic. Percent of control indicates the Growth (OD_600_) or Biofilm (Abs_600_) values for treatment by a given condition divided by the Growth or Biofilm of the matched vehicle control multiplied by 100. The mean Abs_600_ raw values are shown above their respective biofilm bar. Circles show the value of each technical replicate of the triplicate, columns show the mean of each replicate, and the bars show the standard error of the mean. Data points are shown as a percent of control, and the y-axis scale on the left applies to all the graphs in-line. A one-way ANOVA followed by Dunnett’s multiple comparisons test was used to calculate statistical significance in biofilm formation between the untreated control and antibiotic treated wells; * = p value<0.05 ** = p value<0.01, *** = p value <0.001 **** = p value<0.0001.
