# Supplementary material for: Central metabolism is a key player in E. coli biofilm stimulation by sub-MIC antibiotics
Source: PLoS Genet. 2023 Nov 2;19(11):e1011013. doi: 10.1371/journal.pgen.1011013 (PMC10645362; doi:10.1371/journal.pgen.1011013)
Supplement: S2 Fig — Screening results for TET and NOVO shown in a replica plot with extremely high stimulation data points (above 4) removed to help visualize low stimulation data. The data points inside the red box indicate the hits (below 0.5 CR binding). Screening was done in technical duplicate and data from each replicate were plotted on the x and y-axis. (DOCX) [file pgen.1011013.s004.docx]

**
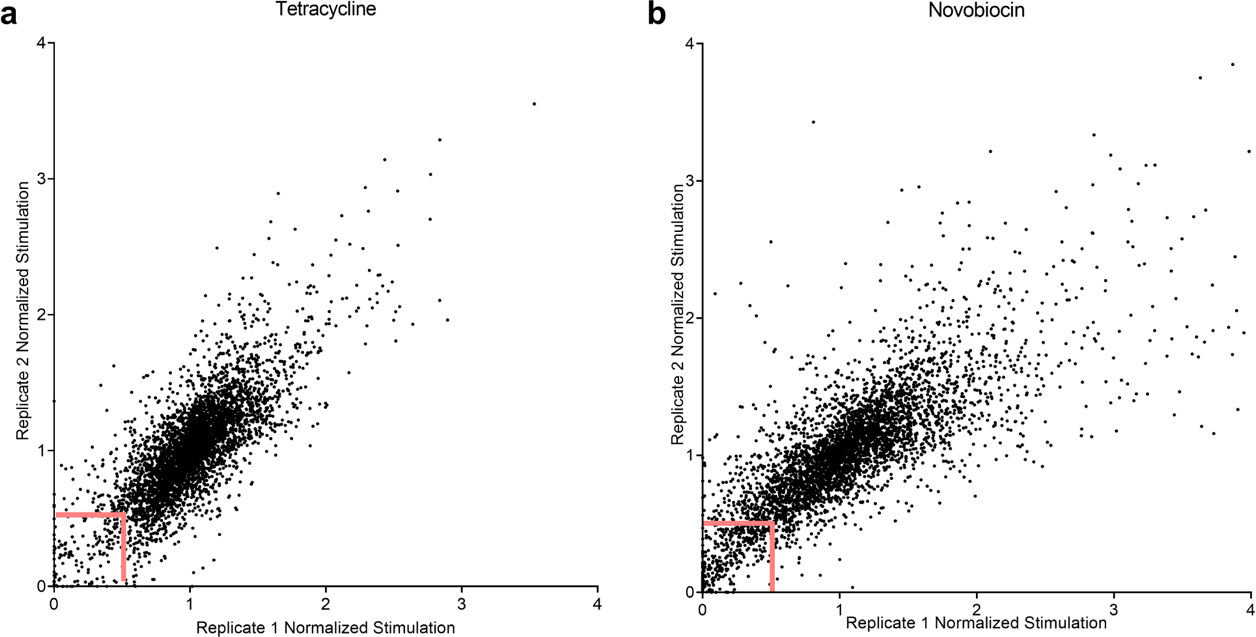
**

**S2 Fig. Biofilm stimulation response to TET and NOVO for the Keio collection.** Screening results for TET (panel a) and NOVO (panel b) shown in a replica plot with extremely high stimulation data points (above 4) removed to help visualize low stimulation data. The data points inside the red box indicate the hits (below 0.5 CR binding). Screening was done in technical duplicate and data from each replicate were plotted on the x and y-axis.
