# Supplementary material for: Central metabolism is a key player in E. coli biofilm stimulation by sub-MIC antibiotics
Source: PLoS Genet. 2023 Nov 2;19(11):e1011013. doi: 10.1371/journal.pgen.1011013 (PMC10645362; doi:10.1371/journal.pgen.1011013)
Supplement: S3 Fig — Hits for each antibiotic condition (below 0.5 CR enrichment and above 0.2 growth) were tabulated and input into a custom Venn diagram generator web-tool (http://bioinformatics.psb.ugent.be/webtools/Venn/) to visualize the data. Numbers enumerate genes that were hits for the indicated antibiotic(s). (DOCX) [file pgen.1011013.s005.docx]

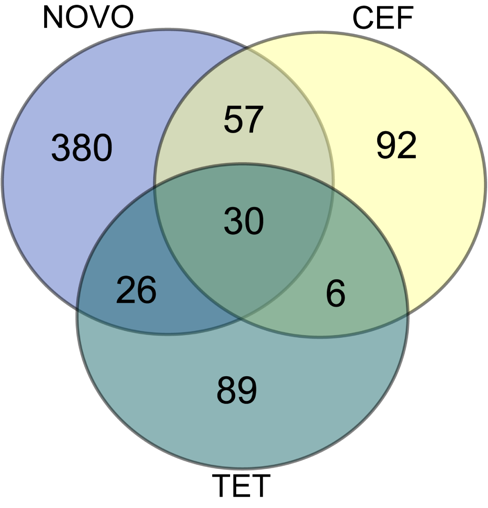


**S3 Fig. Intersection of hits for NOVO CEF or TET.** Hits for each antibiotic condition (below 0.5 CR enrichment and above 0.2 growth) were tabulated and input into a custom Venn diagram generator web-tool (<http://bioinformatics.psb.ugent.be/webtools/Venn/>) to visualize the data. Numbers enumerate genes that were hits for the indicated antibiotic(s).
