# Supplementary material for: Central metabolism is a key player in E. coli biofilm stimulation by sub-MIC antibiotics
Source: PLoS Genet. 2023 Nov 2;19(11):e1011013. doi: 10.1371/journal.pgen.1011013 (PMC10645362; doi:10.1371/journal.pgen.1011013)
Supplement: S4 Fig — The effect of adding 50 mM sodium nitrate on biofilm stimulation was determined by measuring biofilms with a peg lid assay across a range of CEF concentrations. Data points show the growth and biofilm of each well relative to the respective untreated vehicle control (as a percent of control). Percent of control indicates the Growth (OD600) or Biofilm (Abs600) values for treatment by a given condition divided by the Growth or Biofilm of the matched vehicle control multiplied by 100. The mean Abs600 raw values are shown above their respective biofilm bar. The columns show the mean of each technical triplicate, and the error bars show the standard error of the mean. The data are representative of 3 biological replicates. A two-way ANOVA followed by Šidák’s multiple comparisons test was performed in GraphPad Prism to compare biofilm formation between the -/+ nitrate wells (* = p value<0.05, **** = p value <0.0001). Unlabelled columns are all not significant. (DOCX) [file pgen.1011013.s006.docx]

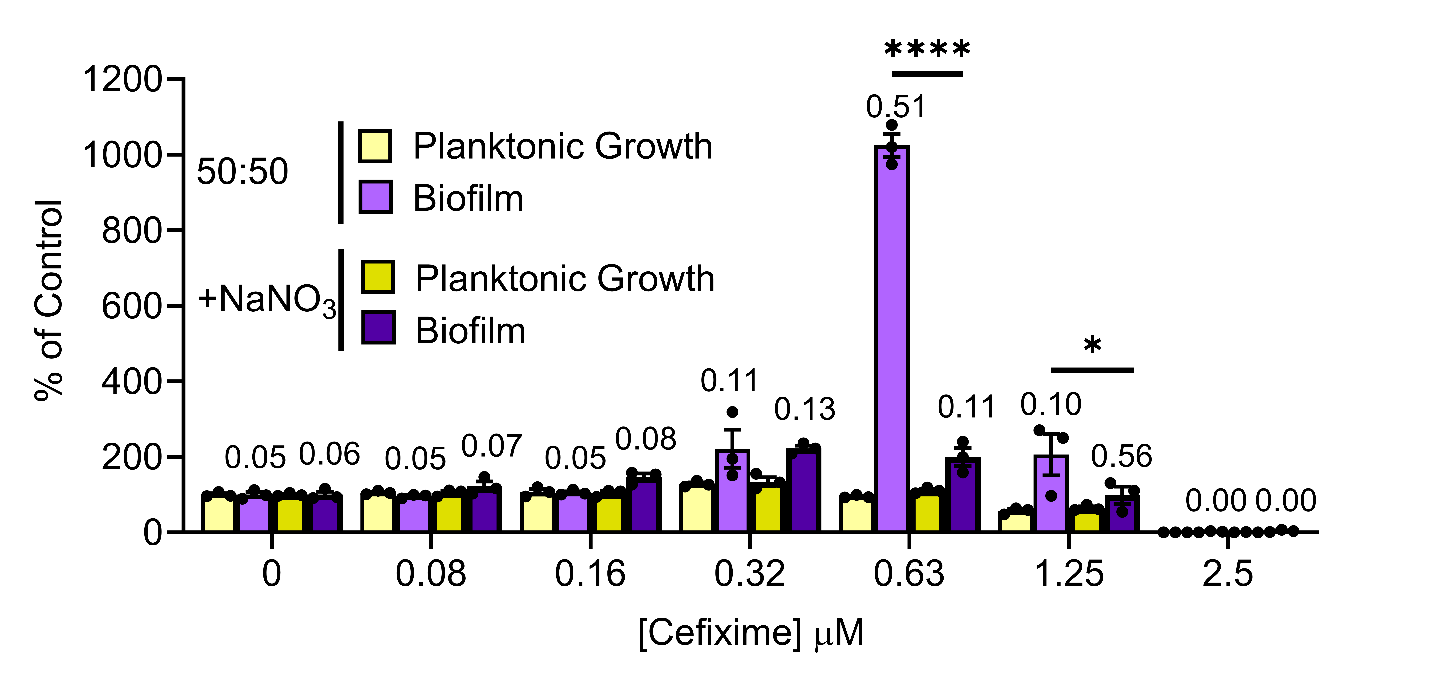


**S4 Fig. Sodium nitrate supplementation suppresses biofilm stimulation.** The effect of adding 50 mM sodium nitrate on biofilm stimulation was determined by measuring biofilms with a peg lid assay across a range of CEF concentrations. Data points show the growth and biofilm of each well relative to the respective untreated vehicle control (as a percent of control). Percent of control indicates the Growth (OD_600_) or Biofilm (Abs_600_) values for treatment by a given condition divided by the Growth or Biofilm of the matched vehicle control multiplied by 100. The mean Abs_600_ raw values are shown above their respective biofilm bar. The columns show the mean of each technical triplicate, and the error bars show the standard error of the mean. The data are representative of 3 biological replicates. A two-way ANOVA followed by Šidák’s multiple comparisons test was performed in GraphPad Prism to compare biofilm formation between the -/+ nitrate wells (* = p value<0.05, **** = p value <0.0001). Unlabelled columns are all not significant.
