# Supplementary material for: Central metabolism is a key player in E. coli biofilm stimulation by sub-MIC antibiotics
Source: PLoS Genet. 2023 Nov 2;19(11):e1011013. doi: 10.1371/journal.pgen.1011013 (PMC10645362; doi:10.1371/journal.pgen.1011013)
Supplement: S6 Fig — Peg lid biofilm assays were performed with increasing concentrations of amikacin, gentamicin, or streptomycin. Growth and biofilm formation are shown as a percent of the vehicle control. Percent of control indicates the Growth (OD600) or Biofilm (Abs600) values for treatment by a given condition divided by the Growth or Biofilm of the matched vehicle control multiplied by 100. The mean Abs600 raw values are shown above their respective biofilm bar. Circles show the value of each technical replicate of the triplicate, columns show the mean of each replicate, and the bars show the standard error of the mean. The graphs are representative of 3 biological replicates. A one-way ANOVA followed by Dunnett’s multiple comparisons test was used to calculate statistical significance in biofilm formation between the untreated control and antibiotic treated wells; none of the antibiotic treatment conditions showed a significant increase in biofilm formation. (DOCX) [file pgen.1011013.s008.docx]

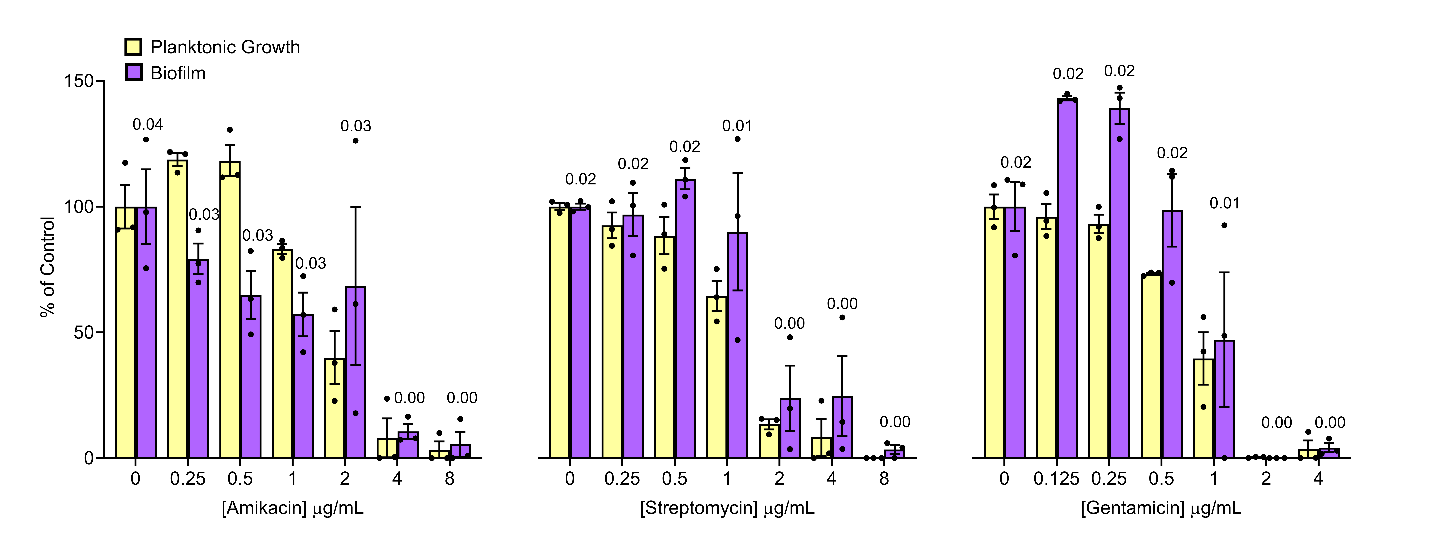


**S6 Fig. Aminoglycosides are poor stimulators of biofilm formation.** Peg lid biofilm assays were performed with increasing concentrations of amikacin, gentamicin, or streptomycin. Growth and biofilm formation are shown as a percent of the vehicle control. Percent of control indicates the Growth (OD_600_) or Biofilm (Abs_600_) values for treatment by a given condition divided by the Growth or Biofilm of the matched vehicle control multiplied by 100. The mean Abs_600_ raw values are shown above their respective biofilm bar. Circles show the value of each technical replicate of the triplicate, columns show the mean of each replicate, and the bars show the standard error of the mean. The graphs are representative of 3 biological replicates. A one-way ANOVA followed by Dunnett’s multiple comparisons test was used to calculate statistical significance in biofilm formation between the untreated control and antibiotic treated wells; none of the antibiotic treatment conditions showed a significant increase in biofilm formation.
